# Modelling, analysis, and stability assessment of wind turbine generator connected to a low inertia AC-DC microgrid with frequency support capability

**DOI:** 10.1038/s41598-025-31517-w

**Published:** 2025-12-23

**Authors:** Islam A. Zenhom, Mostafa I. Marei, Ahmed M. I. Mohamed

**Affiliations:** Department of Electrical Power & Machines, Faculty of Engineering, Ain Shams University, Cairo, 11517 Egypt

**Keywords:** Hybrid grid, Inertia, Stability, Low inertia, Frequency support, Energy science and technology, Engineering

## Abstract

Recently, the integration of renewable energy sources and the development of hybrid AC-DC grids have become increasingly noticeable. Such modern power systems with high penetration of converter-based power sources face many challenges, such as the reduction in the overall system inertia. One of the popular methods to enhance the system’s inertia is to utilize the energy stored in the rotors of wind turbine generators. Although many researchers have proposed effective strategies to address this problem. However, interaction dynamics that might arise between the connected components in a low-inertia AC/DC grid, considering the detailed modelling of each component, are not comprehensively addressed. Therefore, this paper presents a detailed modelling of a typical low-inertia AC/DC grid with frequency support capability offered by a wind generator. The overall system stability is evaluated with the help of the entire system state-space model. Additionally, the influence of varying system parameters on system dominant poles is analyzed to evaluate system stability margins. The study findings are justified through time-domain nonlinear simulations.

## Introduction

Recently, air pollutants that are produced by fossil fuel power stations have been posing a major threat to the environment^1^. The two main factors contributing to environmental pollution are regarded to be global warming and acid rain^2,3^. Due to these issues, governments and other organizations worldwide were compelled to set goals in expanding the use of renewable energy sources (RESs) in the production of electricity as well as the research^4^ provided overview of renewable energy target for 2030 with the united State accounting for 47% of that. With the progression of renewable energy technologies, wind power has substantial growth globally. Unlike conventional power sources, wind energy introduces several areas of exploration, including wind speed forecasting approaches^5,6^ and understanding the dynamic characteristics of wind system. The integration of wind power into the power system has been attracting significant attention in recent times^7–9^, in which frequency and inertia control are studied.

In general, the integration of RESs into a power system diminishes reliance on fossil fuels, enhances voltage profiles, and increases power system reliability^10^. Nonetheless, the extensive incorporation of RESs may introduce significant challenges related to frequency stability^11^. Inertial responses of RESs are usually limited or absent^12^. For instance, power electronic converters are typically used to link variable speed wind turbines to the power grid which decouple the wind turbine inertia from the whole system leading to decreasing the total inertia of the power system. As a consequence of this reduction in inertia, the Rate of Change of Frequency (ROCOF) within the power system may escalate to levels that active the load-shedding controllers, even in instances of minor imbalances. In^13^, a Synchronous Generator (SG) and various RES penetration levels were utilized to meet a load demand. As mentioned in^13^, the power system’s RoCoF tends to rise as the RES penetration level increases. Also, the conventional generating units supporting reserve power decrease with increasing the RESs resulting in increasing the frequency deviation.

Most studies in the literature have discussed the analysis and performance of grid connected wind turbines. In^14,15^ they focused on performance evaluation for grid-connected wind turbine generators. The performances have been simulated and analyzed across four distinct scenarios including varying the wind speed and occurring three phase fault. In^16^, focused on stability enhancement for the wind farm, using a state-space model that crucially incorporates the two-mass wind turbine model to accurately capture torsional dynamics. Furthermore, the study in^17^ addresses low frequency torsional oscillation in a double-fed induction generator wind plant using a damping controller in a battery energy storage. Also, a linearized system model was developed specifically to perform eigenvalue analysis for locating and understanding the oscillatory modes. In^18^, small signal model of wind turbine integrated with power system was studied using the eigenvalues-based method to analyze the influence of control parameters on the power system’s stability. Additionally, proper tuning of the system controllers is essential for optimal operation. In study^19^, the pitch angle controller and the external rotor resistance of a Type-2 wind turbine were optimally tuned using a metaheuristic optimization technique to mitigate low frequency torsional oscillations. The study also employed eigenvalue analysis to evaluate the effectiveness of the proposed controller. Wind turbines’ inertial response can be effectively enhanced by promptly releasing the kinetic energy stored in the rotor over a short period^20,21^. Most inertia controllers employed basic droop or inertia-based strategies, where the turbine’s output power was modulated in proportion to the deviation of system frequency ($\:\varDelta\:f$), its rate of change ($\:df/dt$), or a combination of both, to emulate the inertial behavior of conventional synchronous machines. However, these controllers are often susceptible to noise in the estimated system frequency, which decreases their effectiveness^22^. As a result, a first order filter is integrated into the inertia loop in^23,24^ to eliminate unwanted frequency noise. The study in^25^ presented a small-signal modeling approach for a permanent magnet synchronous generator (PMSG), the nonlinear dynamic model was linearized around an operating point to facilitate stability analysis through eigenvalue evaluation. The effect of varying control parameters—specifically proportional and integral gains—on the system’s eigenvalues was investigated to evaluate their impact on stability. However, the effects of grid and wind turbine parameters were not considered. Also, it did not consider the impact of AC and DC loading which may influence overall system performance in practical applications. In addition, the research^26^ proposed a grid-connected PMSG system and studied variations in system parameters, including inertia and damping, as well as their effects on the system’s eigenvalues. However, it did not consider the two-mass model of the wind turbine or the impact of loading on the system. The limitations of previous studies, which failed to offer a comprehensive analysis of the coupled behavior and the effect of varying most of system parameters on stability margins in addition they did not consider impact of existing DC grid, highlight a critical gap. This paper fills this gap by proposing a comprehensive stability analysis of a PMSG coupled with AC/DC hybrid grid, specifically incorporating the two-mass wind turbine model to fully assess its influence on system damping and stability. Additionally, the research utilizes virtual inertia gain not only for enhanced frequency support but also to specifically study its dynamic effect on the subsystems, including both the DC link and the grid frequency dynamics.

The contributions of this paper can be encapsulated as follows:

- This paper offers a linearized model of a grid connected PMSG wind turbine with an inertial support, taking into account the two-mass model of wind turbine, the interlinking voltage source converter (VSC), the dynamics of the DC link, DC load modelling, AC grid load modeling, and the dynamics of synchronous generator.
- A state-space model for the overall system is derived as well as a stability analysis is presented using eigenvalue-based study to examine how variations in one subsystem (e.g., converter control parameters, shaft stiffness, DC-link characteristics) influence the stability of the others.
- Time domain simulations are provided to validate the system’s performance considering various operating conditions.

**Fig. 1:**
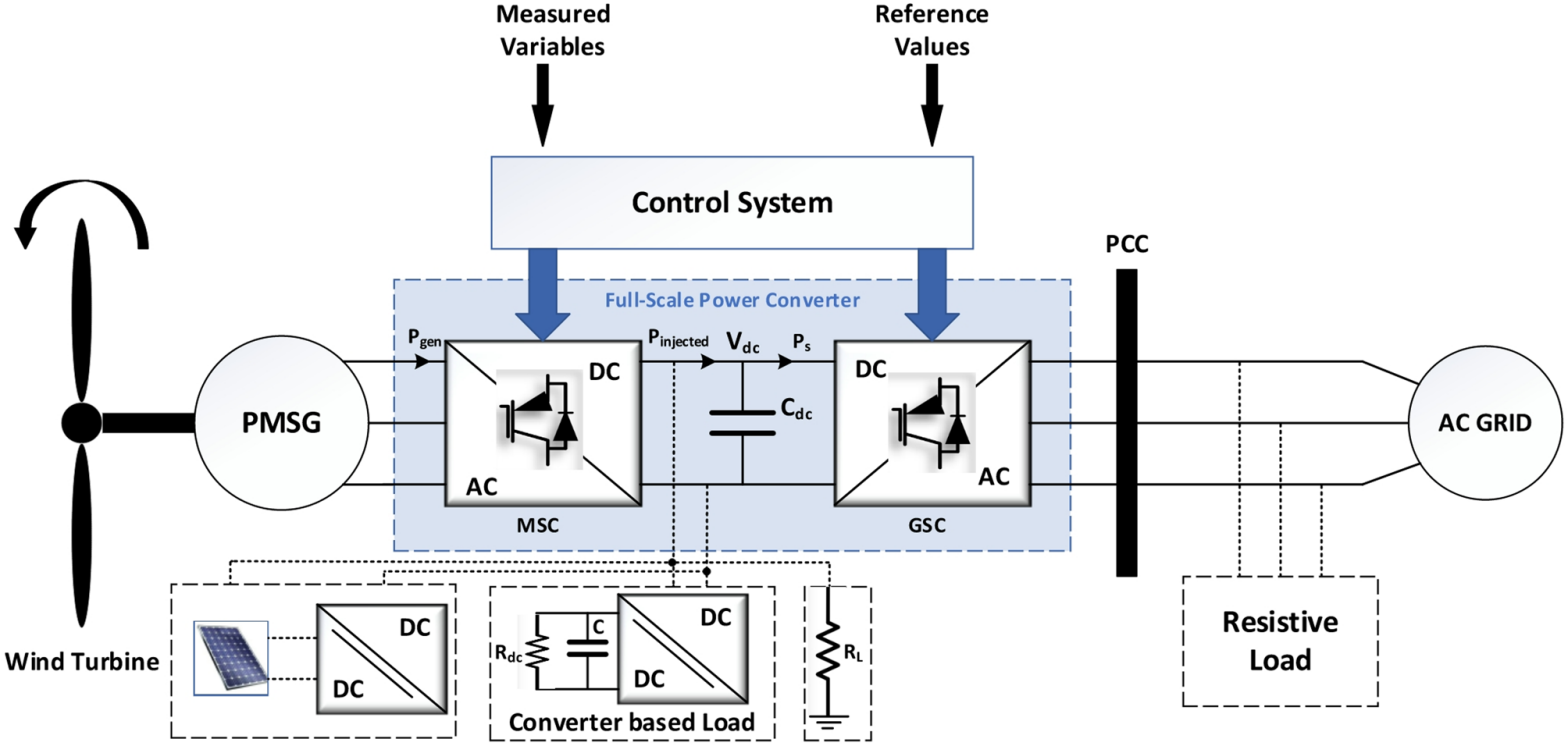
Proposed hybrid grid.

## System description

Figure 1 shows the proposed AC/DC hybrid grid under investigation. A typical AC/DC hybrid grid is comprised of renewable energy sources (RESs), DC loads, resistive loads, and synchronous generators, interfaced to the AC point of common coupling (PCC) and the DC bus. A wind turbine generator based on a PMSG is connected to the hybrid grid through a machine side converter (MSC), which is controlled to inject the maximum available wind power to the hybrid grid. At the DC bus, a DC grid represented by a resistive load, converter-based load, and PV power source, is connected to the hybrid grid. At the PCC, the grid side converter (GSC) interfaces a low-inertia AC grid to the hybrid, where it operates to regulate the DC-link voltage at the DC bus. The function and the model of each component are comprehensively illustrated in the following subsections.

### Grid-side converter (GSC)

The voltage source converter links the RES with the AC grid under investigation. In addition, the VSC is essential for managing power conversion and grid integration in a PMSG wind turbine. It ensures efficient and stable operation by converting the variable frequency and voltage output from the PMSG into a stable AC output that meets grid requirements. It works for power transfer where the VSC converts the AC power of the turbine into DC power, which is then inverted back to AC at the proper voltage and frequency for the electric network as well as employing control approaches to regulate the DC-link voltage and manage both active and reactive power, ensuring system performance and stability. Additionally, the VSC facilitates the integration of the wind turbine with the AC grid, ensuring efficient power transfer and improving stability.

The total power supplied from the DC link by the wind turbine to support the grid ($${P_{injected}})$$ is the result of the generated power of the wind turbine and the net power consumed/absorbed by the DC grid. These dynamics can be represented in (1).

1 $${P_{injected}}={P_{gen}} - \frac{{V_{{dc}}^{2}}}{R} - {P_{CPL}}+{P_{PV}}$$

Where ${P_{gen}}$ is the power generated from the PMSG wind turbine, ${V_{dc}}$ is the dc-link voltage, *R* is the dc resistive load, ${P_{CPL}}$ is the power of converter-based load, ${P_{PV}}$ is the power generated by the PV power source.

The power transferred to the grid ($${P_s}$$) and the dynamics behavior of the dc link capacitor ($${C_{dc}})$$ are defined as:

2 $${P_s}={V_d}{I_{d,VSC}}+{V_q}{I_{q,VSC}}$$

3 $$\frac{1}{2}{C_{dc}}V_{{dc}}^{2}={P_{injected}} - {P_s}$$

Where the ${V_d}$, ${V_q}$ are d-axis and q-axis grid voltages. ${I_{d,VSC}}$, ${I_{q,VSC}}$ are d-axis and q-axis output currents of the VSC, respectively.

The VSC control loop dynamics are represented in Eqs. (4–8), where $I_{d}^{*},~I_{q}^{*}$ are the reference values of d-q currents, respectively. ${k_{vp}},{k_{vi}}$ are the proportional and integral gains of the VSC’s outer power loop, respectively. $\tau$ is the current controller time constant. Assuming the reference q-axis current $I_{q}^{*}$ is zero is when operating the system at unity power factor. This assumption implies that the VSC only supplies active power, with no reactive power exchange. So, it minimizes the reactive current component, which in turn reduces the total current magnitude flowing through the converter and associated components. This reduction in current results in lower conduction losses in the system, improving overall efficiency.

4 $$I_{d}^{*}=\frac{1}{{1.5~{V_d}}}\left( {\left( {V_{{dc,ref}}^{2} - V_{{dc}}^{2}} \right)\left( {{k_{vp}}+\frac{{{k_{vi}}}}{s}} \right)+{P_s}} \right)$$

5 $$I_{q}^{*}=0$$

6 $${I_{d,VSC}}=\frac{{I_{d}^{*}}}{{1+~\tau {\text{~s}}}}$$

7 $${P_{injected}}={P_{gen}} - \frac{{V_{{dc}}^{2}}}{R} - {P_{CPL}}+{P_{PV}}$$

8 $${I_{q,VSC}}=\frac{{I_{q}^{*}}}{{1+~\tau {\text{~s}}}}$$

### PMSG wind turbine

The dynamics of the PMSG wind turbine, including the behavior of both the wind turbine and the synchronous generator are detailed in^27^.

- Wind turbine model.

Based on the wind turbine characteristics:

9 $${P_w}=\frac{1}{2}\rho A{C_p}(\lambda ,\beta ){\nu ^3}$$

10 $$\lambda =\frac{{{\omega _r}r}}{\nu },A=\pi {r^2}$$

where $\rho$ is the air density (kg/m^3^), *A* is the blade area (m^2^), ${C_p}$ is the power coefficient, $\lambda$ is the ratio of tip speed, $\beta$ is the pitch angle(deg.), $\nu$ is the wind speed (m/s), *r* is the blade radius, and ${\omega _r}$ is the turbine rotor speed (rad/s).

The wind turbine should be operated at the most economical operating point to extract the maximum power. It can be achieved by maintaining the rotor speed at its optimal value at the optimal tip speed ratio.

11 $${P_{opt}}=\frac{1}{2}\rho A{C_{p,opt}}~{\left( {\frac{r}{{{\lambda _{opt}}}}} \right)^3}~\omega _{{r,opt}}^{3}$$

Where ${P_{opt}}$, ${\lambda _{opt}}$, ${C_{p,opt}}$, ${\omega _{r,opt}}$, represent optimal power, tip speed, power coefficient, and rotor speed, respectively.

Also, the two-mass system of the wind turbine is represented as follows^28^:

12 $$\frac{{d{\omega _{tur}}}}{{dt}}=\frac{1}{{2H}}\left( {{T_{tur}} - {T_{sh}}} \right)$$

13 $$\frac{{d\theta }}{{dt}}={\omega _{base}}\left( {{\omega _{tur}} - {\omega _r}} \right)$$

14 $${T_{sh}}={K_s}\theta +D\left( {{\omega _{tur}} - {\omega _r}} \right)$$

Where ${\omega _{tur}}$, ${\omega _r}$ are the turbine speed and generator speed. ${T_{tur}}$, ${T_{sh}}$ are the turbine and shaft torque. ${K_s}$, *D*, $\theta$, *H* are the shaft stiffness, damping factor, shaft angle, and inertia constant, respectively.

**Fig. 2:**
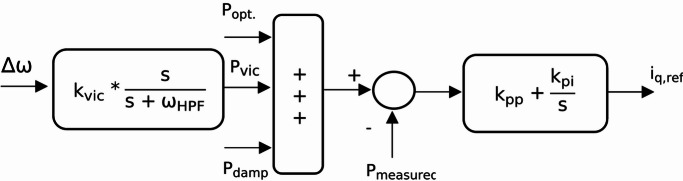
Power control loop for PMSG wind turbine.

To aid in frequency regulation, additional active power must be drawn from the wind turbine through inertia control during disturbances. Inertia control ensures that the wind turbine can respond quickly to changes in grid frequency, providing a crucial buffer during unexpected disturbances. Additionally, research such as^29^ has proposed a damping power to mitigate oscillations and mechanical stress caused by the two-mass mechanical system. By damping these oscillations, the system can operate more smoothly and efficiently, reducing wear on mechanical components and enhancing overall reliability. This integration of inertia control and damping power is essential for the robust operation of wind turbines in dynamic environments. So, Fig. 2 show the reference power using high pass filter (HPF) inertia controller. This reference generated power ( ${P_{g,ref}}$) is defined in Eq. (15) where ${P_{vic}}$ and ${P_{damp}}$ are the generated power from inertia control and damping power defined in Eqs. (16,17), respectively. $\Delta \omega$ is the grid angular frequency deviations, ${K_{vic}}$ is the inertia gain, ${K_{damp}}$ is the damping gain, ${G_{bpf\left( s \right)}}$ is the band pass filter transfer function, and ${k_{pp}}$, ${k_{pi}}$ are the proportional and integral gains of the MSC’s outer power loop, respectively.

15 $${P_{g,ref}}={P_{opt}}+{P_{vic}}+{P_{damp}}$$

16 $${P_{vic}}=\left( {{k_{vic}}*\frac{s}{{s+{\omega _{HPF}}}}} \right)\Delta \omega$$

17 $${P_{damp}}={G_{bpf\left( s \right)}}*{k_{damp}}*\Delta \omega$$

- PMSG model.

The dynamic model of the PMSG in d-q reference frame is defined in the following equations:

18 $${V_{ds}}={i_{ds}}{r_s}+{L_d}\frac{{d{i_{ds}}}}{{dt}} - {\omega _r}p{L_q}{i_{qs}}$$

19 $${V_{qs}}={i_{qs}}{r_s}+{L_q}\frac{{d{i_{qs}}}}{{dt}}+{\omega _r}p{L_d}{i_{ds}}+{\omega _r}p{\lambda _m}$$

20 $${T_g}=\frac{3}{2}p\left( {{\lambda _m}{i_{qs}}+({L_d} - {L_q}} \right){i_{ds}}{i_{qs}})$$

21 $${T_{sh}} - {T_g} - B{\omega _r}=J\frac{{d{\omega _r}}}{{dt}}$$

Where (${V_{ds}}$, ${V_{qs}}$) and (${i_{ds}}$, ${i_{qs}}$) are the d-q representation of stator voltages and currents. (${L_d}$, ${L_q}$) are d-q axes components of winding inductance, ${r_s}$ is the stator winding resistance and ${\lambda _m}$ is the magnetic flux. *p* is the number of pair poles, *B* is the generator damping coefficient, *J* is the generator inertia, and ${T_g}$ is the electro- magnetic torque.

### Low-inertia AC grid model

**Fig. 3:**
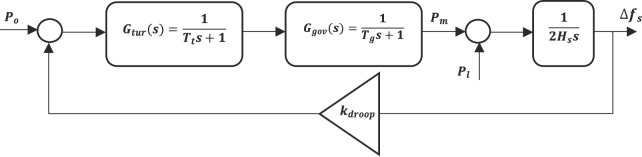
Automatic generation control for AC grid.

In this model, the AC electric grid is represented by a collection of parallel-connected synchronous generators, each equipped with droop control. Due to the droop control implementation, power sharing among the generators is achieved naturally, with each unit contributing power according to its droop coefficient. The generator has governor and turbine which are modelled according to the swing equation and is depicted in Fig. 3.

22 $${P_m} - {P_l}=2{H_s}s\Delta {f_s}$$

23 $${P_m}={G_{gov}}\left( s \right){G_{tur}}\left( s \right)\left\{ {{P_o} - {k_{droop}}\Delta {f_s}} \right\}$$

24 $${G_{gov}}\left( s \right)=\frac{1}{{{T_g}s+1}}$$

25 $${G_{tur}}\left( s \right)=\frac{1}{{{T_t}s+1}}$$

Where ${P_m}$, ${P_l}$, and ${P_o}$ are the mechanical power, demand power, and reference generated power, respectively. ${H_s}$ and ${k_{droop}}$ are the grid inertia and droop gain. ${f_s}$ is the system frequency. ${G_{gov}}\left( s \right)$ and ${G_{tur}}\left( s \right)$ are the governor and turbine controller each having a time delay of ${T_g}$ and ${T_t}$. $\Delta {f_s}$ is the deviation in system frequency.

## System state-space model

The previous systems’ equations are represented in a state space form using the approach described in^30,31,32^. It is shown that the system is portioned to subsystems which are modelled separately, and each subsystem can be represented as a set of non-linear equations as follows:

26 $${\dot {x}_n}={f_n}\left( {{x_n},{u_n}} \right)$$

27 $${y_n}={g_n}\left( {{x_n},{u_n}} \right)$$

Where ${x_n}$, ${u_n}$, and ${y_n}$ are the $nth$ state variables, input variables, and output variables of the substate space form.

These non-linear equations are linearized and represented in small signal state space form as follows:

28 $$\Delta {\dot {x}_n}={a_n}~\Delta {x_n}+{b_n}~\Delta {u_n}$$

29 $$\Delta {y_n}={c_n}~\Delta {x_n}+{d_n}~\Delta {u_n}$$

Where ${a_n}$, ${b_n}$, ${c_n}$, and ${d_n}$ are the subsystem model matrices. For instance, to represent the PMSG in state space form, small signal variable should be defined, $\Delta {i_{ds}}$, $\Delta {i_{qs}}$, $\Delta {\omega _r}$. Then, linearized the electrical and mechanical Eqs. (18–21) as follows:

30 $$\Delta {V_{ds}}=\Delta {i_{ds}}{r_s}+{L_d}\frac{{d\Delta {i_{ds}}}}{{dt}} - {\omega _{ro}}p{L_q}\Delta {i_{qs}} - \Delta {\omega _r}p{L_q}{i_{qso}}$$

31 $$\Delta {V_{qs}}=\Delta {i_{qs}}{r_s}+{L_q}\frac{{d\Delta {i_{qs}}}}{{dt}}+{\omega _r}p{L_d}\Delta {i_{ds}}+\Delta {\omega _r}p{L_d}{i_{ds0}}+\Delta {\omega _r}p{\lambda _m}$$

32 $$\Delta {T_g}=\frac{3}{2}p\left( {{\lambda _m}\Delta {i_{qs}}+\left( {{L_d}} \right. - {L_q}} \right)\left. {\left( {{i_{dso}}\Delta {i_{qs}}+{i_{qso}}\Delta {i_{ds}}} \right)} \right)$$

33 $$\Delta {T_m} - \Delta {T_g} - B\Delta {\omega _r}=J\frac{{d\Delta {\omega _r}}}{{dt}}$$

where $\Delta$ means the small perturbation and ${i_{dso}}$, ${i_{qso}}$ are the steady state values for d-q stator current components. Then, re-arrange the equations to the standard form to represent in state-space model.

34 $$\frac{{d\Delta {i_{ds}}}}{{dt}}= - \frac{{{r_s}}}{{{L_d}}}\Delta {i_{ds}}~+\frac{{p{\omega _{ro}}{L_q}}}{{{L_d}}}\Delta {i_{qs}}+\frac{{p{L_q}{i_{qso}}}}{{{L_d}}}\Delta {\omega _r}+\frac{1}{{{L_d}}}\Delta {V_{ds}}$$

35 $$\frac{{d\Delta {i_{qs}}}}{{dt}}= - \frac{{{r_s}}}{{{L_q}}}\Delta {i_{qs}} - \frac{{p{\omega _r}{L_d}}}{{{L_q}}}\Delta {i_{ds}} - \left( {\frac{{{L_d}{i_{dso}}}}{{{L_q}}}+\frac{{{\lambda _m}}}{{{L_q}}}} \right)p\Delta {\omega _r}+\frac{1}{{{L_q}}}\Delta {V_{qs}}$$

36 $$\frac{{d\Delta {\omega _r}}}{{dt}}=\frac{1}{J}\Delta {T_m} - \frac{1}{J}\Delta {T_g} - \frac{B}{J}\Delta {\omega _r}$$

37 $${T_g}=\frac{3}{2}p\left( {\left( {{\lambda _m}+({L_d} - {L_q}){i_{dso}}} \right)\Delta {i_{qs}}+{{\left( {{L_d} - L} \right.}_q}} \right){i_{qso}}\left. {\Delta {i_{ds}}} \right)$$

According to Eqs. (28,29), build the state space form as follows:

38 $$\left[ {\begin{array}{*{20}{c}} {\mathop {\Delta {i_{ds}}}\limits^{.} } \\ {\mathop {\Delta {i_{qs}}}\limits^{ \cdot } } \\ {\mathop {\Delta {\omega _r}}\limits^{ \cdot } } \end{array}} \right]={a_1}\left[ {\begin{array}{*{20}{c}} {\Delta {i_{ds}}} \\ {\Delta {i_{qs}}} \\ {\Delta {\omega _r}} \end{array}} \right]+{b_1}~\left[ {\begin{array}{*{20}{c}} {\Delta {V_{ds}}} \\ {\Delta {V_{qs}}} \\ {\Delta {T_{sh}}} \end{array}} \right]$$

39 $$\left[ {\begin{array}{*{20}{c}} {{i_{ds}}} \\ {{i_{qs}}} \\ {{\omega _r}} \\ {{T_g}} \\ {{P_g}} \end{array}} \right]={c_1}\left[ {\begin{array}{*{20}{c}} {\Delta {i_{ds}}} \\ {\Delta {i_{qs}}} \\ {\Delta {\omega _r}} \end{array}} \right]+{d_1}~~\left[ {\begin{array}{*{20}{c}} {\Delta {V_{ds}}} \\ {\Delta {V_{qs}}} \\ {\Delta {T_{sh}}} \end{array}} \right]$$

Where,

40 $${a_1}=\left[ {\begin{array}{*{20}{c}} { - \frac{{{r_s}}}{{{L_d}}}}&{\frac{{p{\omega _{ro}}{L_q}}}{{{L_d}}}}&{\frac{{p{L_q}{i_{qso}}}}{{{L_d}}}} \\ { - \frac{{p{\omega _r}{L_d}}}{{{L_q}}}}&{ - \frac{{{r_s}}}{{{L_q}}}}&{ - \left( {\frac{{{L_d}{i_{dso}}}}{{{L_q}}}+\frac{{{\lambda _m}}}{{{L_q}}}} \right)p} \\ { - \frac{3}{{2J}}p({L_d} - {L_q}){i_{qso}}}&{ - \frac{3}{{2J}}p({\lambda _m}+({L_d} - {L_q}){i_{dso}})}&{ - \frac{B}{J}} \end{array}} \right]$$

41 $${b_1}=\left[ {\begin{array}{*{20}{c}} {\frac{1}{{{L_d}}}}&0&0 \\ 0&{\frac{1}{{{L_q}}}}&0 \\ 0&0&{\frac{1}{J}} \end{array}} \right],~~$$

42 $${c_1}=\left[ {\begin{array}{*{20}{c}} 1&0&0 \\ 0&1&0 \\ 0&0&1 \\ {\frac{3}{2}p({L_d} - {L_q}){i_{qso}}}&{\frac{3}{2}p({\lambda _m}+({L_d} - {L_q}){i_{dso}})}&0 \\ { - \frac{3}{2}{V_d}}&{ - \frac{3}{2}{V_q}}&0 \end{array}} \right]$$

43 $${d_1}=\left[ {\begin{array}{*{20}{c}} 0&0&0 \\ 0&1&0 \\ 0&0&1 \\ {\frac{3}{2}p({L_d} - {L_q}){i_{qso}}}&{\frac{3}{2}p({\lambda _m}+({L_d} - {L_q}){i_{dso}})}&0 \\ { - \frac{3}{2}{V_d}}&{ - \frac{3}{2}{V_q}}&0 \end{array}} \right]$$

Where ${P_g}$ is the generator PMSG power. This identical procedure (modeling in state-space form) is applied to all other subsystems, including the wind turbine, converter controls, and AC grid. Then, there are procedures to get the overall state space system model^33^. The first step is the interconnections between the sub-models that are described by the following equations:

44 $$\Delta u={L_1}~\Delta y+{L_2}~\Delta {u_{sys}}$$

45 $$\Delta {y_{sys}}={L_3}~\Delta y+{L_4}~\Delta {u_{sys}}$$

**Fig. 4:**
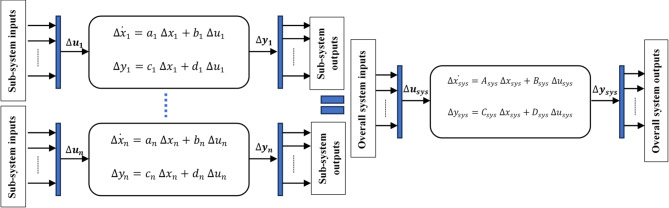
Combining the state-space sub-models to get overall system state space model.

Where $\Delta u$ and $\Delta y$ are the resulted vectors of the input and output vectors of all sub-models in Eqs. (28,29). $\Delta {u_{sys}}$ and $\Delta {y_{sys}}$ are the input and output vectors for the overall system. ${L_1}$, ${L_2}$, ${L_3}$, and ${L_4}~$represents the matrices that show the interconnections between the sub-models. The second step is formulating an entire system from all sub-models, as shown in Fig. 4.

46 $$\left[ {\begin{array}{*{20}{c}} {\mathop {\Delta {x_1}}\limits^{ \cdot } } \\ \vdots \\ {\mathop {\Delta {x_n}}\limits^{ \cdot } } \end{array}} \right]=\left[ {\begin{array}{*{20}{c}} {{a_1}}&{}&{} \\ {}& \ddots &{} \\ {}&{}&{{a_n}} \end{array}} \right]~\left[ {\begin{array}{*{20}{c}} {\Delta {x_1}} \\ \vdots \\ {\Delta {x_n}} \end{array}} \right]+\left[ {\begin{array}{*{20}{c}} {{b_1}}&{}&{} \\ {}& \ddots &{} \\ {}&{}&{{b_n}} \end{array}} \right]~\left[ {\begin{array}{*{20}{c}} {\Delta {u_1}} \\ \vdots \\ {\Delta {u_n}} \end{array}} \right]$$

47 $$\left[ {\begin{array}{*{20}{c}} {\Delta {y_1}} \\ \vdots \\ {\Delta {y_n}} \end{array}} \right]=\left[ {\begin{array}{*{20}{c}} {{c_1}}&{}&{} \\ {}& \ddots &{} \\ {}&{}&{{c_n}} \end{array}} \right]~\left[ {\begin{array}{*{20}{c}} {\Delta {x_1}} \\ \vdots \\ {\Delta {x_n}} \end{array}} \right]+\left[ {\begin{array}{*{20}{c}} {{d_1}}&{}&{} \\ {}& \ddots &{} \\ {}&{}&{{d_n}} \end{array}} \right]~\left[ {\begin{array}{*{20}{c}} {\Delta {u_1}} \\ \vdots \\ {\Delta {u_n}} \end{array}} \right]$$

Then, the overall state space system model should be:

48 $$\mathop {\Delta {x_{sys}}}\limits^{ \cdot } ={A_{sys}}~\Delta {x_{sys}}+{B_{sys}}~\Delta {u_{sys}}$$

49 $$\Delta {y_{sys}}={C_{sys}}~\Delta {x_{sys}}+{D_{sys}}~\Delta {u_{sys}}$$

Where

50 $${A_{sys}}=a+b~{L_1}{\left( {I - d~{L_1}} \right)^{ - 1}}c$$

51 $${B_{sys}}=b~{L_1}{\left( {I - d~{L_1}} \right)^{ - 1}}d~{L_2}+b~{L_2}$$

52 $${C_{sys}}=~{L_3}{\left( {I - d~{L_1}} \right)^{ - 1}}c$$

53 $${D_{sys}}={L_3}{\left( {I - d~{L_1}} \right)^{ - 1}}d~{L_2}+{L_4}$$

Where *a, b, c* and *d* are the matrices presented in Eqs. (46,47). In addition, ${A_{sys}}$, ${B_{sys}}$, ${C_{sys}}$, and ${D_{sys}}$ are the overall system model matrices which the system eigenvalues can be extracted from them.

## Model validation

**Fig. 5:**
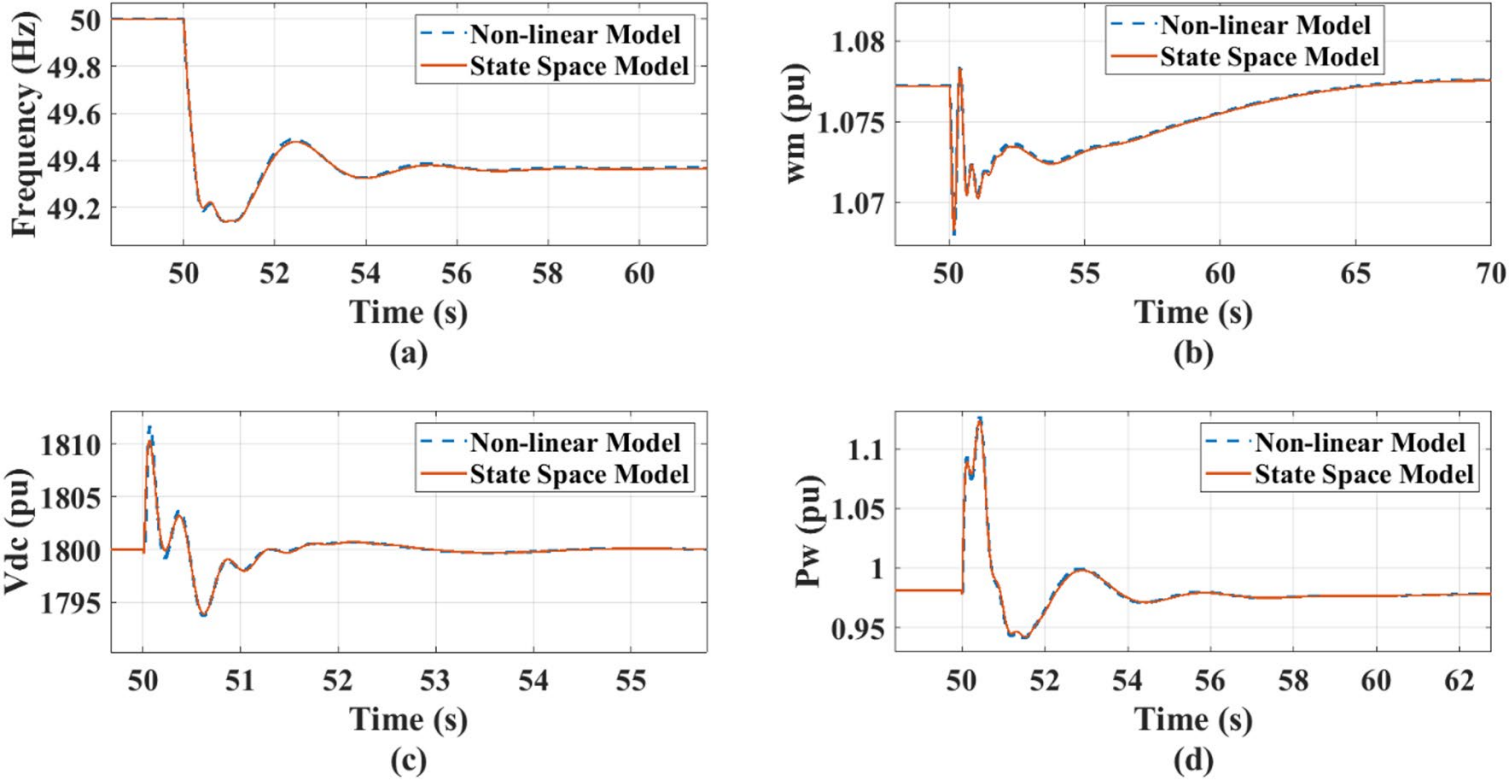
Comparison between the system non-linear model and proposed state-space model: (a) Grid frequency response, (b) Wind rotational speed response, (c) DC- link voltage response, (d) Wind output power.

The system dynamic response and its overall system’s state space are employed on the MATLAB/ Simulink library. The overall small-signal state space model dynamics are compared with the non-linear model of the Simulink model in MATLAB. The system’s operating point is set for a wind speed of 12 m/s operating at Maximum Power Point (MPP) producing 2 MW, the optimum tip speed ratio is 8.1, and the rotating speed is 2.7636 rad/s. The DC-link voltage is maintained at 1800v, supplies a 1 MW DC load (resistive/CPL), while the grid with 1.5s inertia constant, operating at 690v, is simultaneously supporting with the PMSG a 3 MW static AC load. The PMSG model’s steady state d-axis current ${i_{dso}}$ is 0 and its q-axis current ${i_{qso}}$ is 0.6672 pu. Figure 5 presents a comparison between the dynamic behavior of nonlinear system model and the corresponding linearized state-space model evaluated at the same operating points and conditions. The disturbance is introduced on the AC side at time *t* = 50 s by increasing the load on the AC side by 0.5 MW. The dc-link voltage, grid frequency, and rotational speed of the PMSG turbine verify that the two models have very close dynamics, where both models accurately capture the system’s fast and slow transients. This confirms that the linearized system can be effectively used to assess the system stability margins and examine the effects of the system parameters’ variations.

## Stability analysis

This section analyzes the system’s stability under variations in different network parameters and the effect of adding outer damping loop on the system. The system parameters provided in Table 1 are considered the base values of the system under investigation. These nominal values are used as reference points for parameter variation. The next subsections illustrate the impact of varying different system parameters on the system poles. Also, the controllers’ gains are presented in Table 2. Since the system is simulated in per unit, most gains are unitless.

**Table 1:** System parameters used in the proposed model.

Parameter	Value	Parameter	Value
Rated voltage	690 V	DC link capacitance	24 mf
AC grid rated power	2 MVA	Resistive load	1 MW
Rated frequency	50 Hz	AC static load	3 MW
Grid inertia (${H_s}$)	1.5 s	Wind speed	12 m/s
DC link voltage	1800 V	PMSG rated power	2 MW
Magnetic flux ($${\lambda _m}$$)	9.5474 Wb	Number of pair poles ($$p)$$	32

**Table 2:** Controllers’ gains.

Gain	${k_{pp}}$	${k_{pi}}$	${k_{vp}}$	${k_{vi}}$	${k_{damp}}$	${k_{droop}}$	${k_{vic}}$	${k_s}$
Nominal value [Pu]	0.002	100	5	100	80	20	25	1.6

### Virtual inertia gain variation

**Fig. 6:**
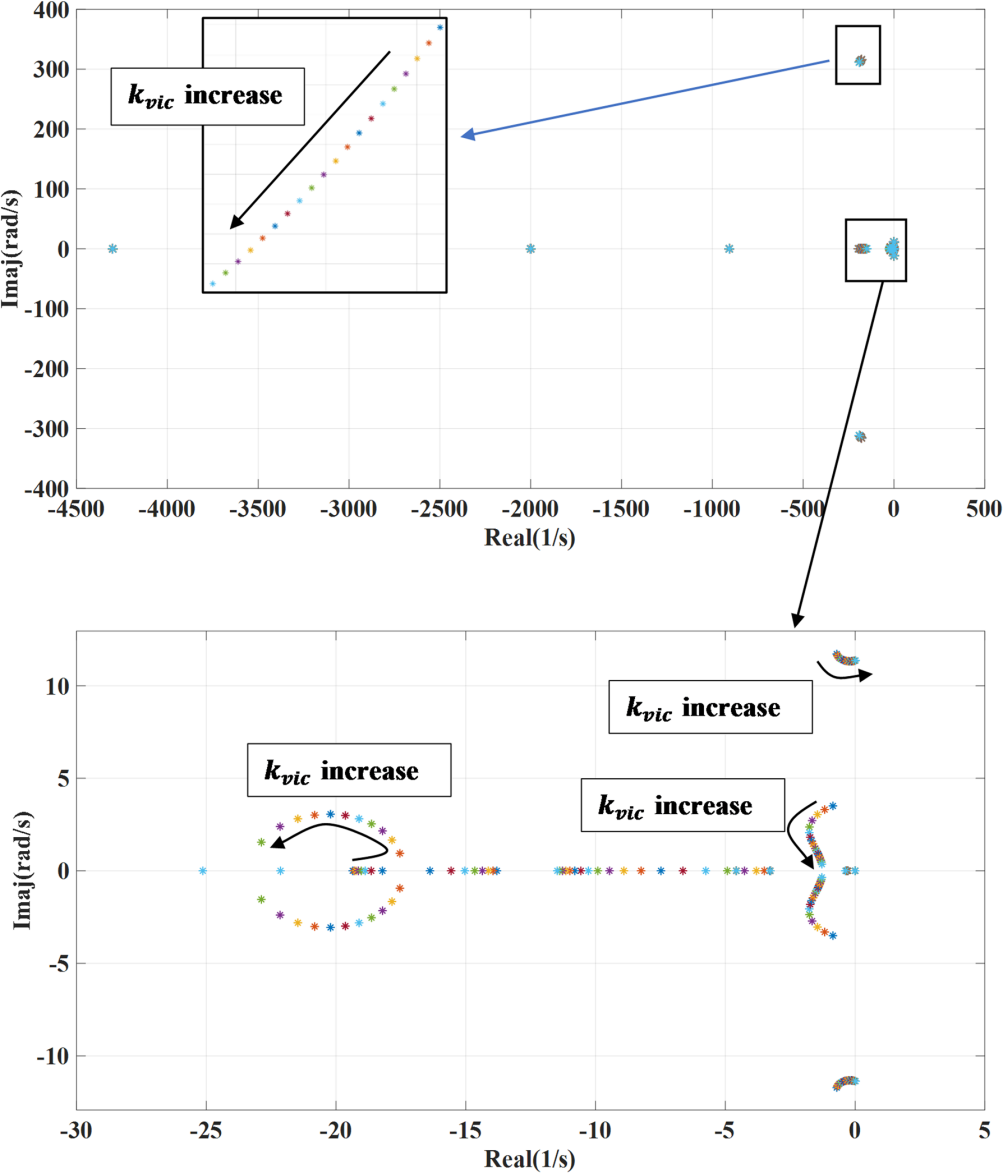
System eigenvalues variation against virtual inertia gain with deactivating outer damping loop.

**Fig. 7:**
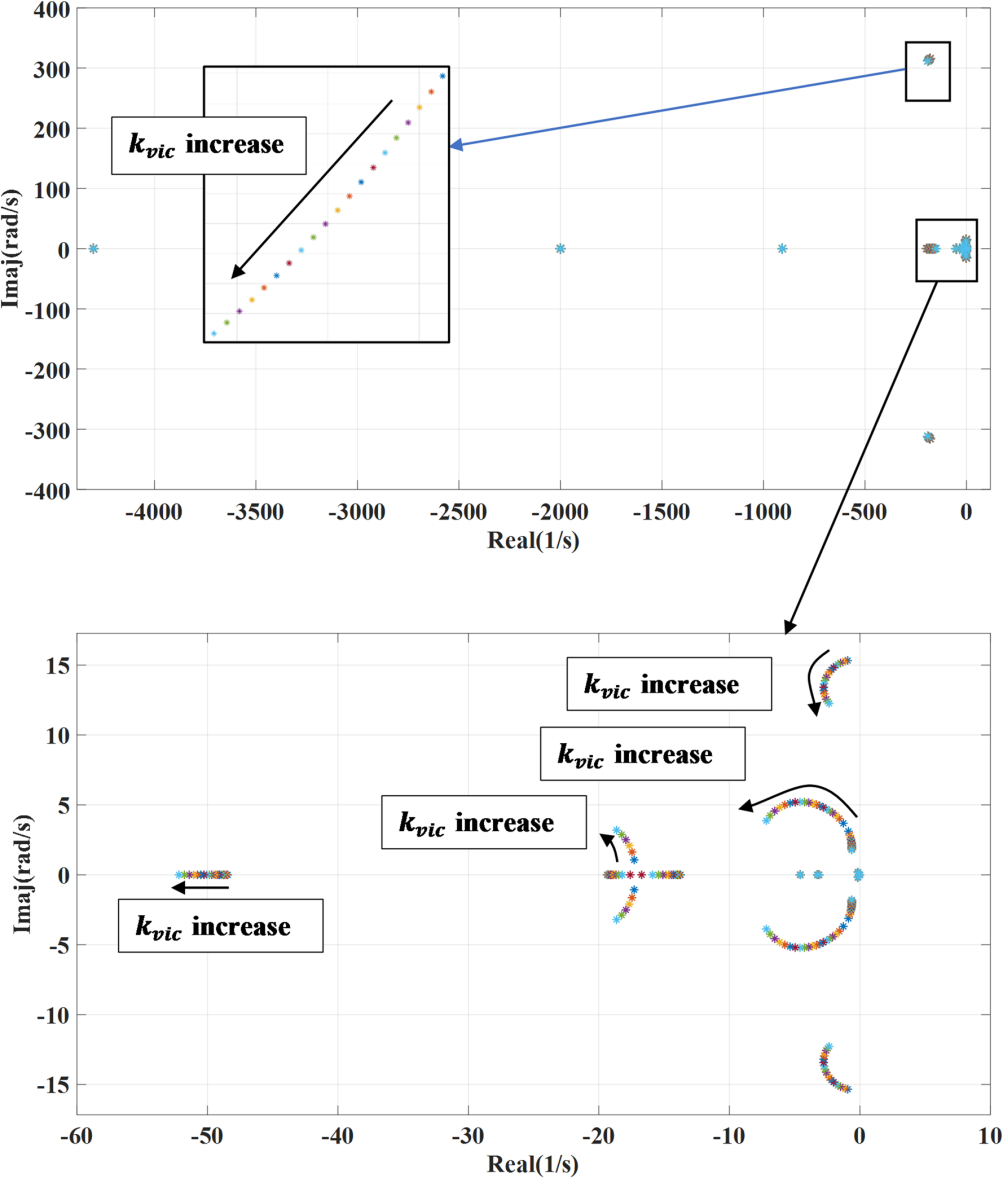
System eigenvalues variation against virtual inertia gain with activating outer damping loop.

Figure 6 portrays the impact of changing the virtual inertia gain ($\:{K}_{vic}$) on the systems’ eigenvalues. This gain is changed from 0.1 to 2 of the nominal value while deactivating the damping loop of the wind turbine generator converter. Increasing the virtual inertia gain typically shifts the system’s eigenvalues further into the left side, meaning their real parts become more negative. This shift reflects enhanced damping and system stability. However, the eigenvalues linked to fast dynamics move toward the right side suggesting a slower dynamic response. An increase in virtual inertia gain strengthens the coupling between the system and grid frequency, which can slow down the transient response. However, excessive gain may ultimately lead to system instability. When activating outer damping loop as shown in Fig. 7, with increasing the virtual inertia gain, the systems’ eigenvalues shift toward the left side enhancing the stable response. The combination of outer damping and virtual inertia supports more damping to the system improving the ability to deal with oscillations and disturbance. This will result in lower risk of instability and better frequency regulation.

### Shaft stiffness variation of the PMSG wind turbine

**Fig. 8:**
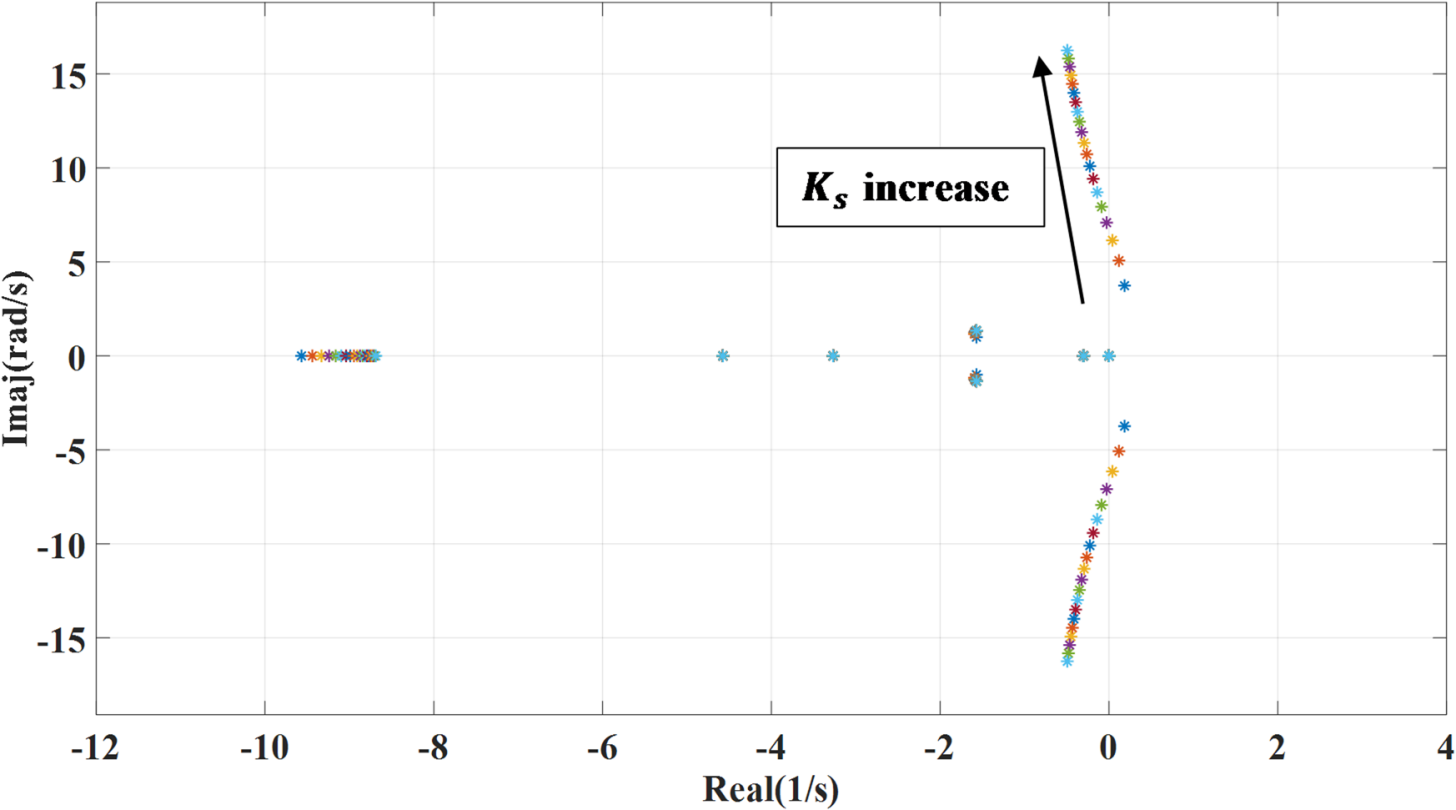
System eigenvalues variation against wind turbine shaft stiffness with deactivating outer damping loop.

**Fig. 9:**
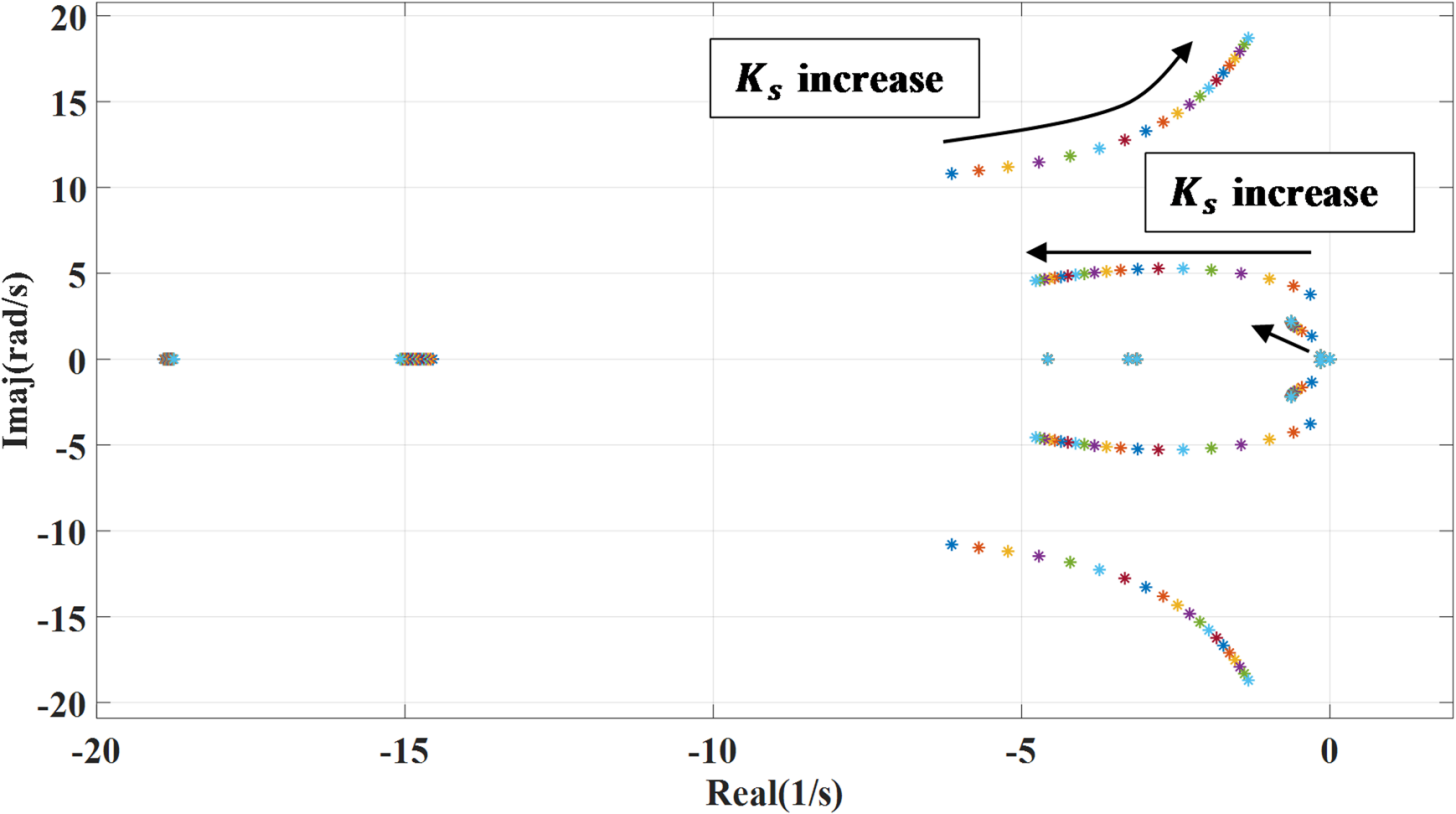
System eigenvalues variation against wind turbine shaft stiffness with activating outer damping loop.

Figures 8 and 9 show the impact of changing the shaft stiffness ($\:{k}_{s}$) on the dominant systems’ eigenvalues. The gain is changed from 0.1 to 2 of the nominal value. while deactivating the outer damping loop, as the shaft stiffness increases, the systems’ poles move slightly leftward indicating more damping and stability lead to a more rigid coupling between turbine and generator. However, at the lower shaft stiffness, the system introduces low frequency torsional oscillations which may interact with the control system causing instability as shown. Compared to the previous case, with adding the outer damping loop, the systems’ poles shift leftward to a more stable region, suppressing oscillations and enhancing damping.

### The outer loop controller bandwidth

**Fig. 10:**
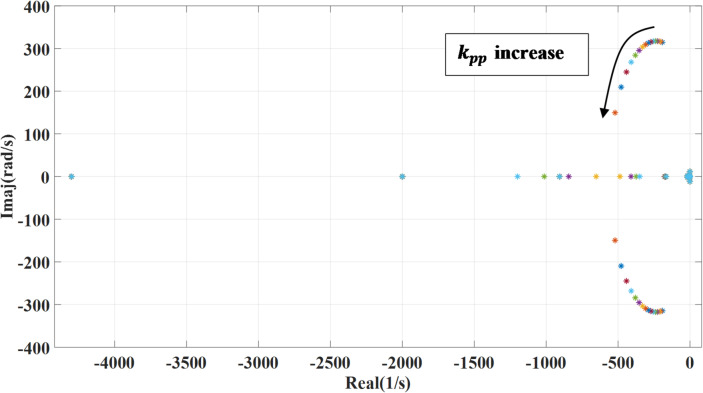
System eigenvalues variation against proportional gain $\:{k}_{pp}$.

**Fig. 11:**
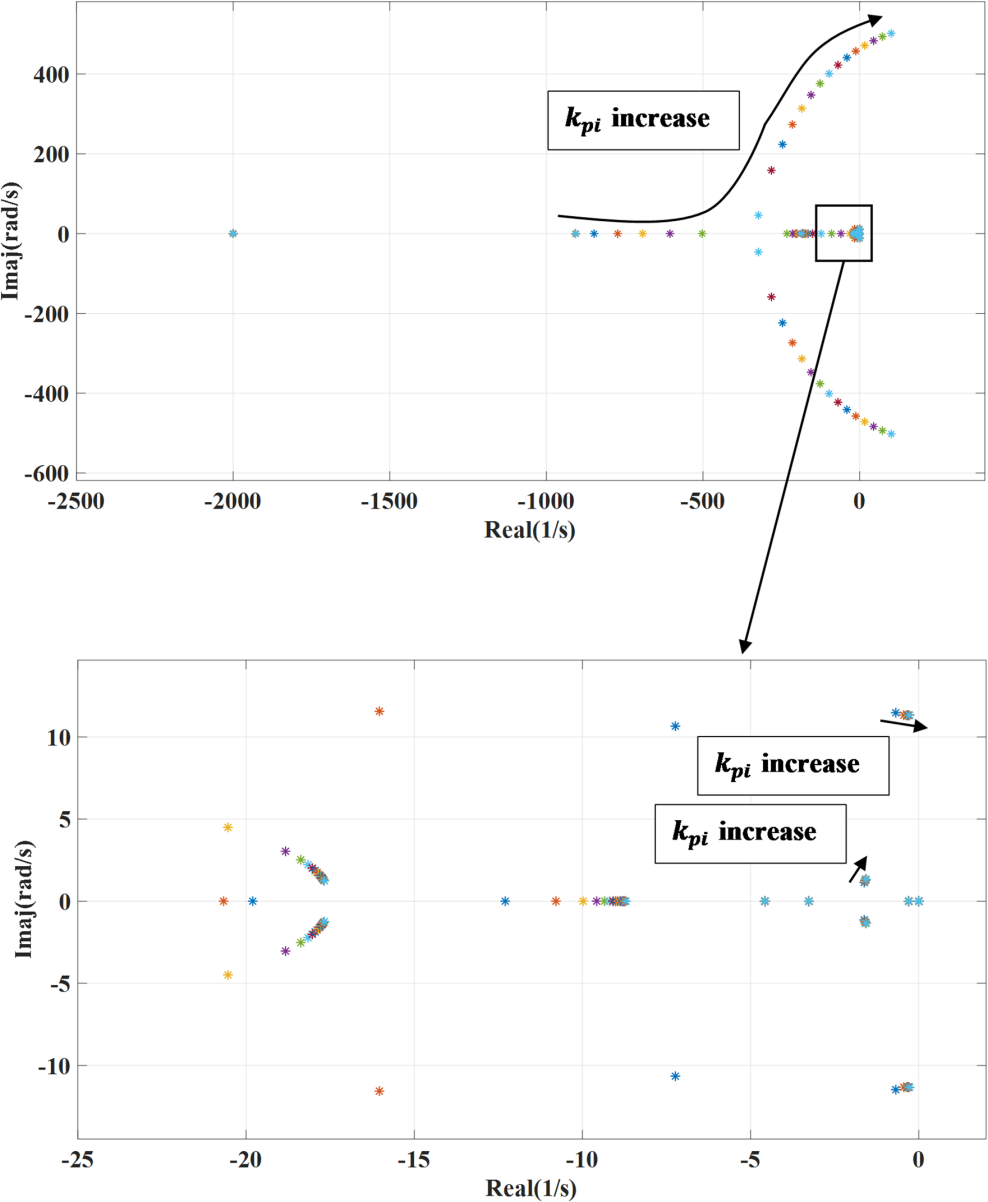
System eigenvalues variation against integral gain $\:{k}_{pi}$ with deactivating outer damping loop.

**Fig. 12:**
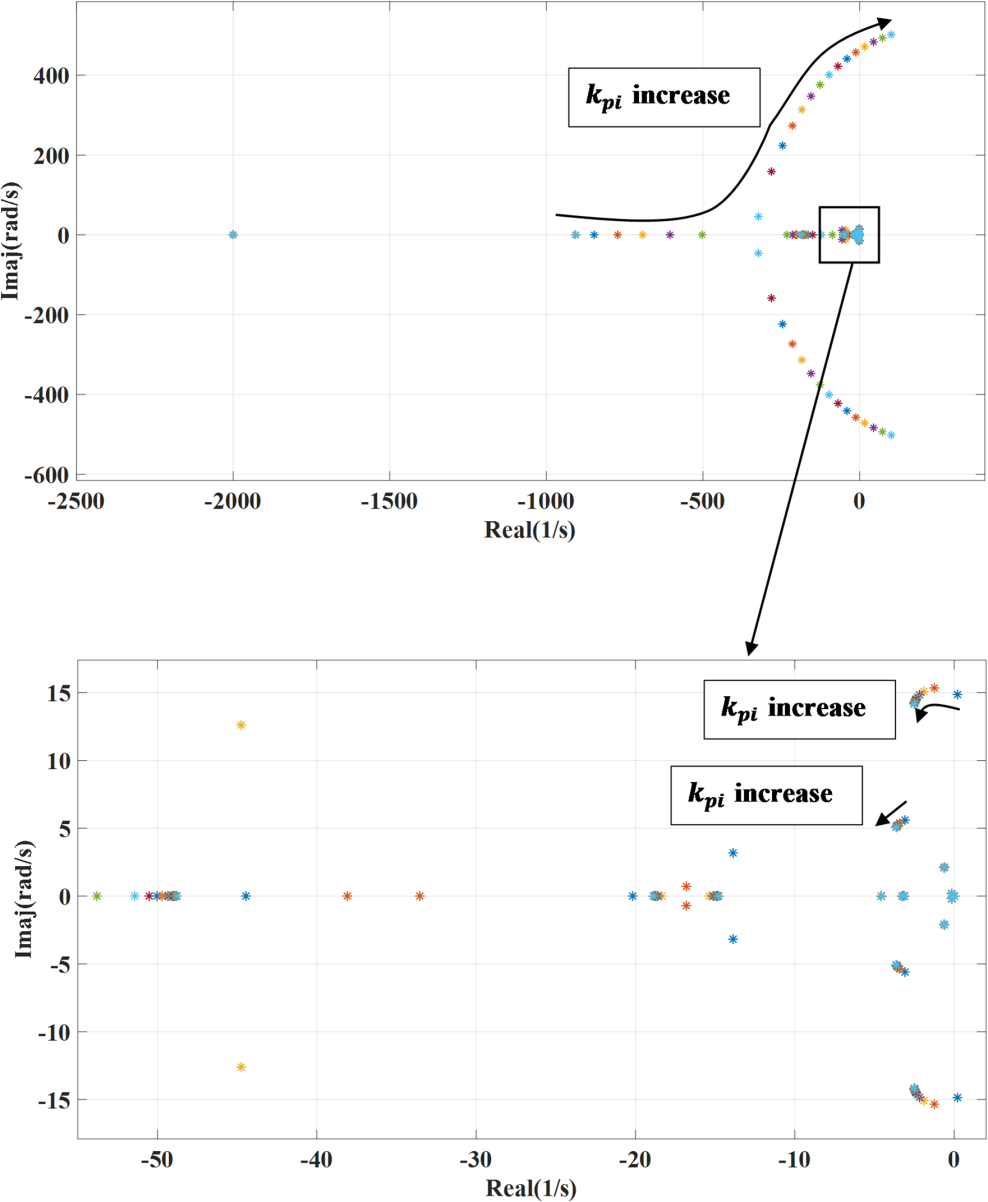
System eigenvalues variation against integral gain $\:{k}_{pi}$ with activating outer damping loop.

The machine side converter outer loop controller bandwidth represented in terms of the proportional and integral gain, the following Fig. 10 shown the impact of changing proportional gain ($\:{k}_{pp}$) of the outer loop power controller on the systems’ eigenvalues. Increasing the proportional gain enhances the system’s response to errors, allowing it to correct deviations more rapidly. As $\:{k}_{pp}$ increases, the real parts of the system’s eigenvalues shift further to the left on the complex plane, indicating improved damping and a more stable response. With activating the outer damping loop, there will be no effect on the previous response during changing the proportional gain. Also, the effect of varying the integral gain is presented in Figs. 11 and 12 which show the impact of changing integral gain ($\:{k}_{pi}$) of the outer loop power controller on the dominant systems’ eigenvalues and it also shows the effect of activating damping power on the variation. The integral gain is changed from 0.1 to 20 of the nominal value. While the integral gain helps eliminate steady-state error, it can adversely affect the system’s damping where increasing the integral gain can shift the system’s eigenvalues closer to the imaginary axis, which reduces damping and may lead to increased oscillations or slower settling times, depending on the system dynamics. To overcome the negative effect caused by integral gain, the additional damping enhances the system stability and increases damping, reducing the potential underdamping effects caused by the integral gain.

### DC-link capacitance variation

**Fig. 13:**
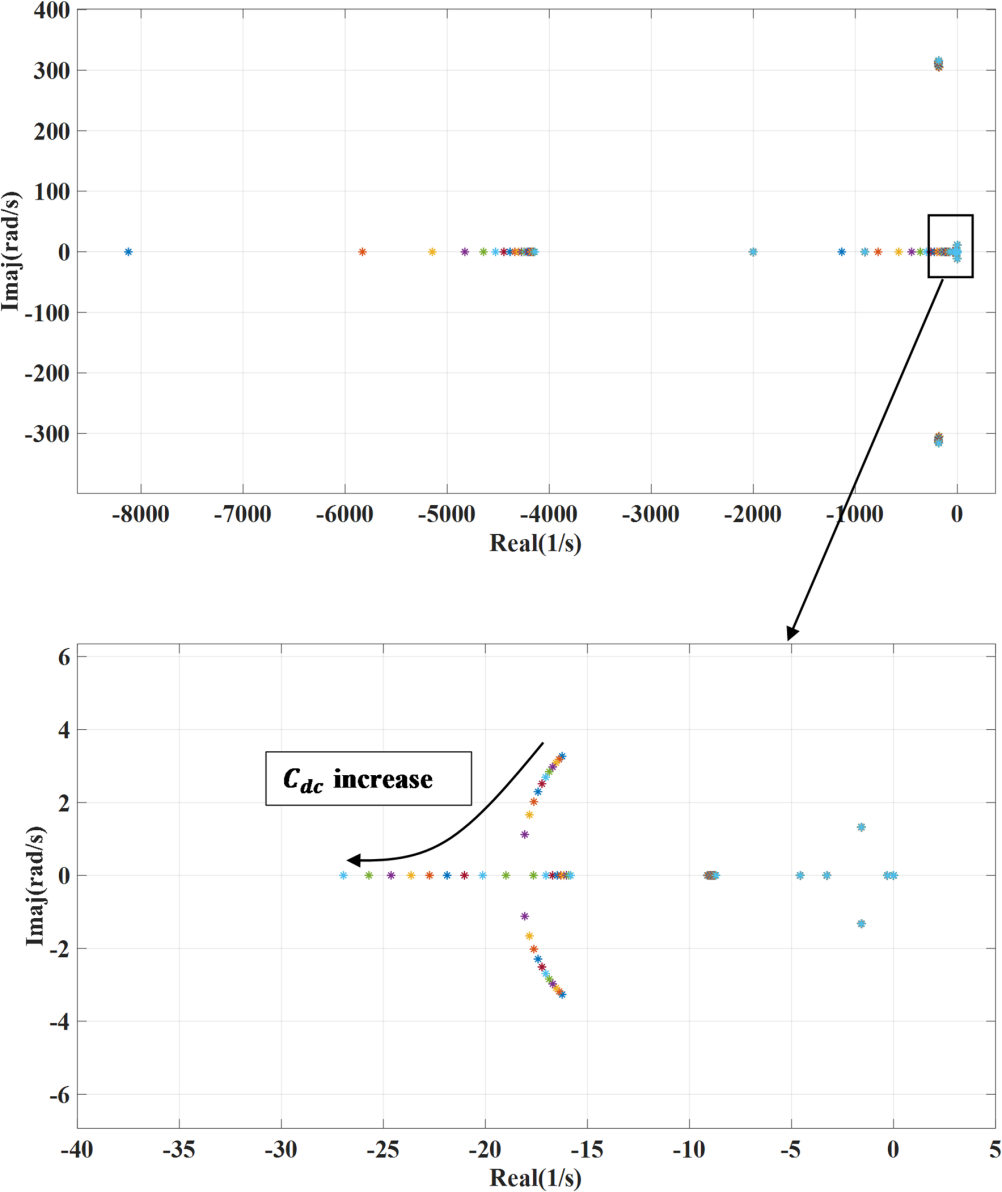
System eigenvalues variation against dc-link capacitance with deactivating outer damping loop.

**Fig. 14:**
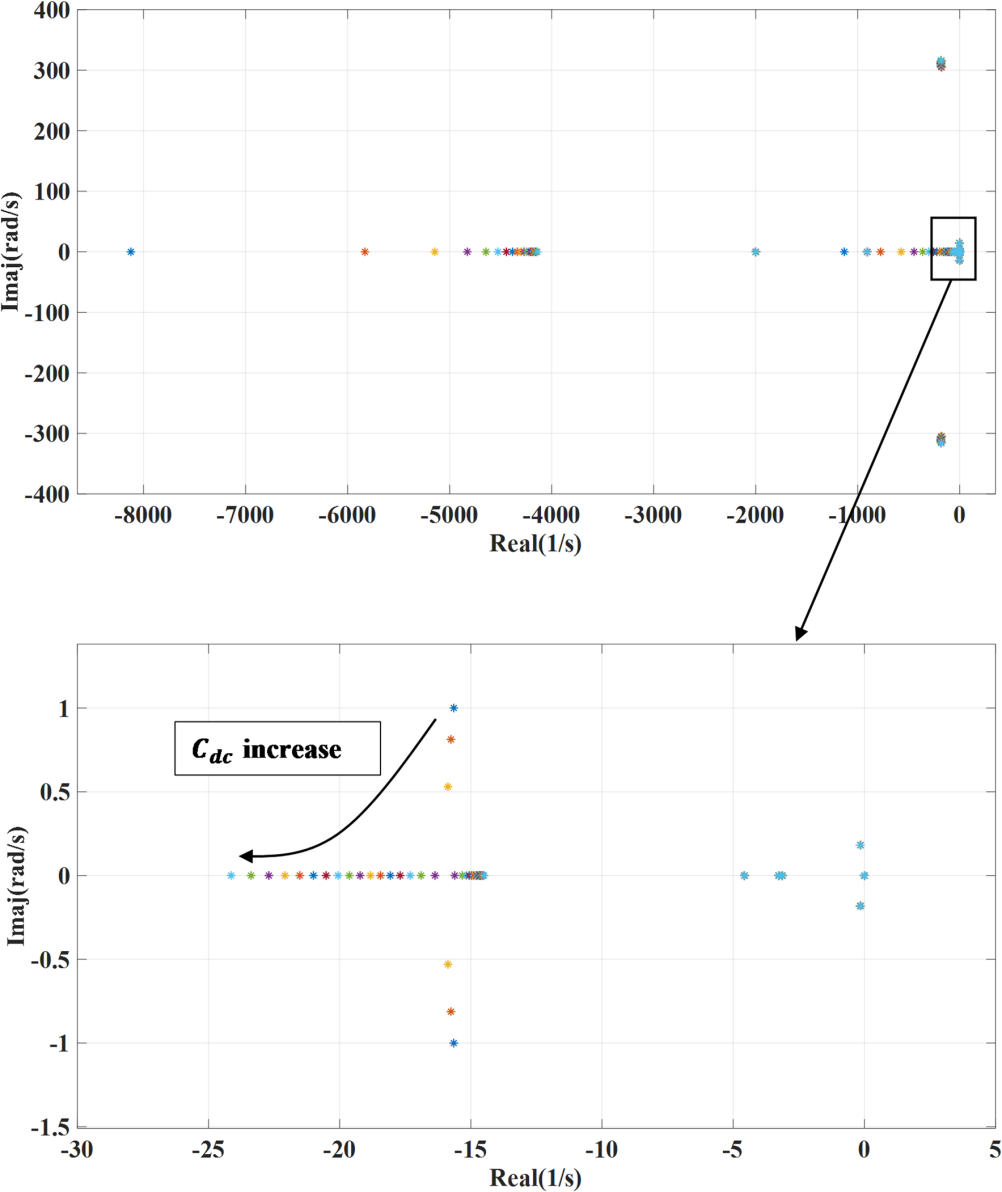
System eigenvalues variation against dc-link capacitance with activating outer damping loop.

Figure 13 presents the impact of changing the dc link capacitance ($\:{C}_{dc}$) on the systems’ eigenvalues. This parameter is changed from 0.1 to 2 of the design value while deactivating the damping loop. By increasing the dc-link capacitance, the energy storage capability of the system is enhanced, which tends to reduce voltage fluctuations and dampen high-frequency oscillations. This, in turn, shifts the system’s eigenvalues toward the left half of the complex plane, indicating improved damping and increased stability margins as shown. However, the combined effect of adding an outer damping loop and changing the DC link capacitance enhances both damping and system stability while the outer damping loop contributes by actively mitigating low-frequency oscillations as shown in Fig. 14.  However, to achieve optimal performance, careful tuning is required, as excessive capacitance may slow down system response.

### Inertia constant variation

**Fig. 15:**
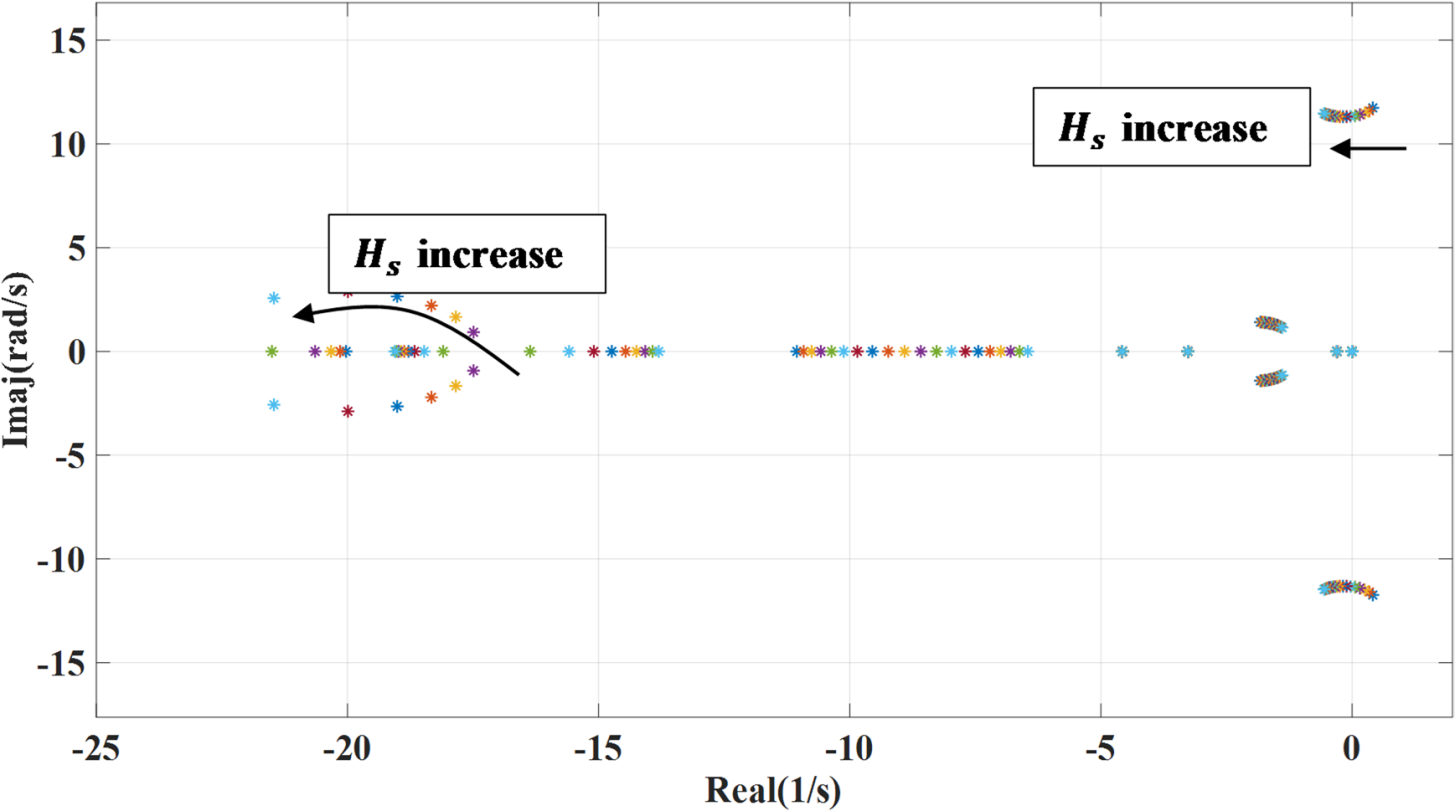
System eigenvalues variation against grid inertia constant with deactivating outer damping loop.

**Fig. 16:**
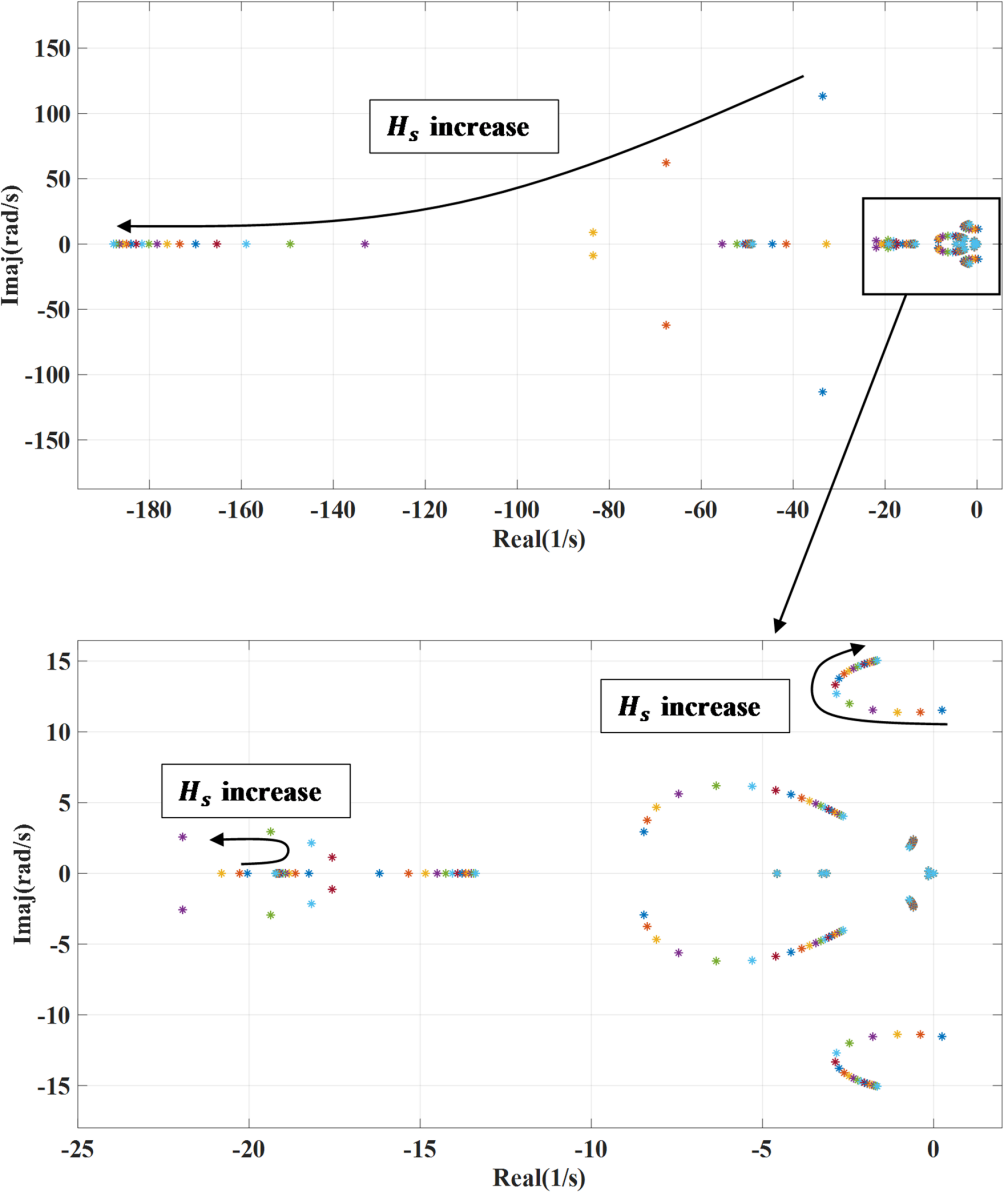
System eigenvalues variation against grid inertia constant with activating outer damping loop.

Figures 15 and 16 demonstrate the impact of changing the inertia constant ($\:{H}_{s}$) on the dominant systems’ eigenvalues. The inertia constant is changed from 0.1 to 2 of the nominal value. In case of no outer damping power, lower grid inertia affects on the systems’ poles by moving closer to imaginary part leading to unstable behavior, as illustrated in Fig. 15 that make the system more sensitive to disturbances. However, with increasing the inertia constant, the systems’ poles shift from the unstable region toward the stable region, and the system damping is enhanced. Thus, the system becomes less sensitive to disturbances. Also, lower inertia combined with outer damping enables the system to respond more quickly and enhances overall stability. In contrast, when grid inertia is high, the system becomes more resistant to rapid changes and may not require additional damping. However, introducing damping in such cases can be beneficial, as it helps the system settle faster and suppress oscillations.

### Impact of DC grid load type

**Fig. 17:**
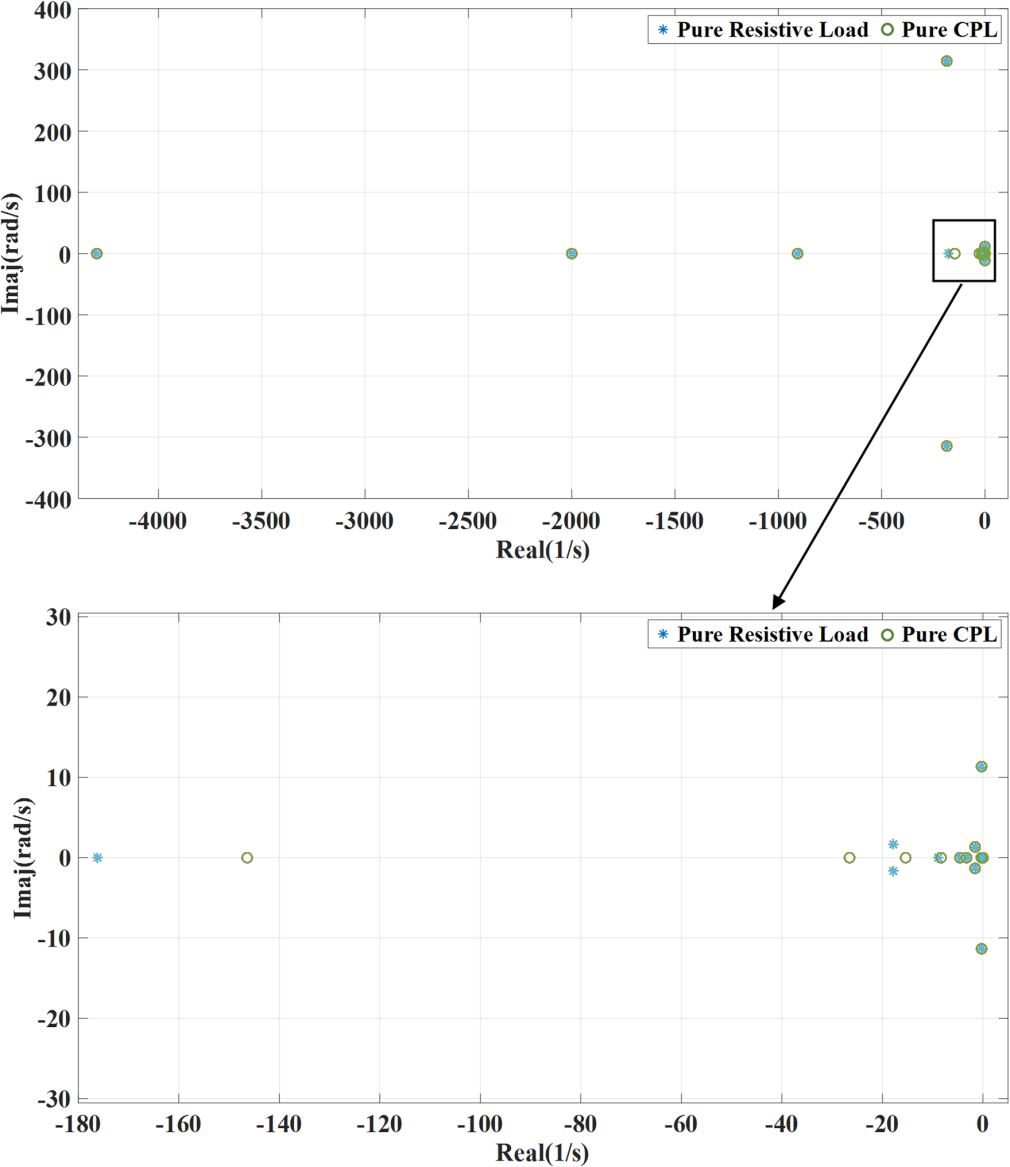
Eigenvalues at resistive and constant power load types.

Figure 17 illustrates the variation of the system’s eigenvalues for two different loading conditions: a DC resistive load and a CPL both connected on the DC-link side. The presence of the CPL has a relatively small impact on the system’s dynamic behavior, particularly its damping characteristics. As shown in the figure, the eigenvalues of the system shift slightly to the right in the complex plane when a CPL is used, compared to the resistive load. This rightward shift indicates a minor reduction in system damping. In contrast, the resistive load maintains better damping performance, with poles located further to the left, suggesting a more stable and well-damped response.

Based on the eigenvalue analysis, the following Table 3 summarizes the system parameters categorized by their level of influence on overall stability, ranging from low to high impact.

**Table 3:** System parameters’ impact levels.

Parameters	Impact level	Parameters	Impact level	Parameters	Impact level
Wind shaft stiffness ${K_s}$	High	Virtual inertia gain ${K_{vic}}$	Moderate	DC link capacitance ${C_{dc}}$	Low
Machine Side Converter Outer loop bandwidth	High	Grid inertia constant ${H_s}$	Moderate	DC grid load type	Low

## Simulation results

The system’s dynamic response should reflect the behavior of its eigenvalues as system parameters vary. This section presents simulation results to validate the theoretical findings and further investigate the impact of varying key system parameters on dynamic performance. By subjecting the system to step changes and disturbances under different parameter settings, the transient and steady-state behavior is observed and analyzed. These simulations help confirm the correlation between eigenvalue shifts and time-domain responses.

### Virtual inertia gain

**Fig. 18:**
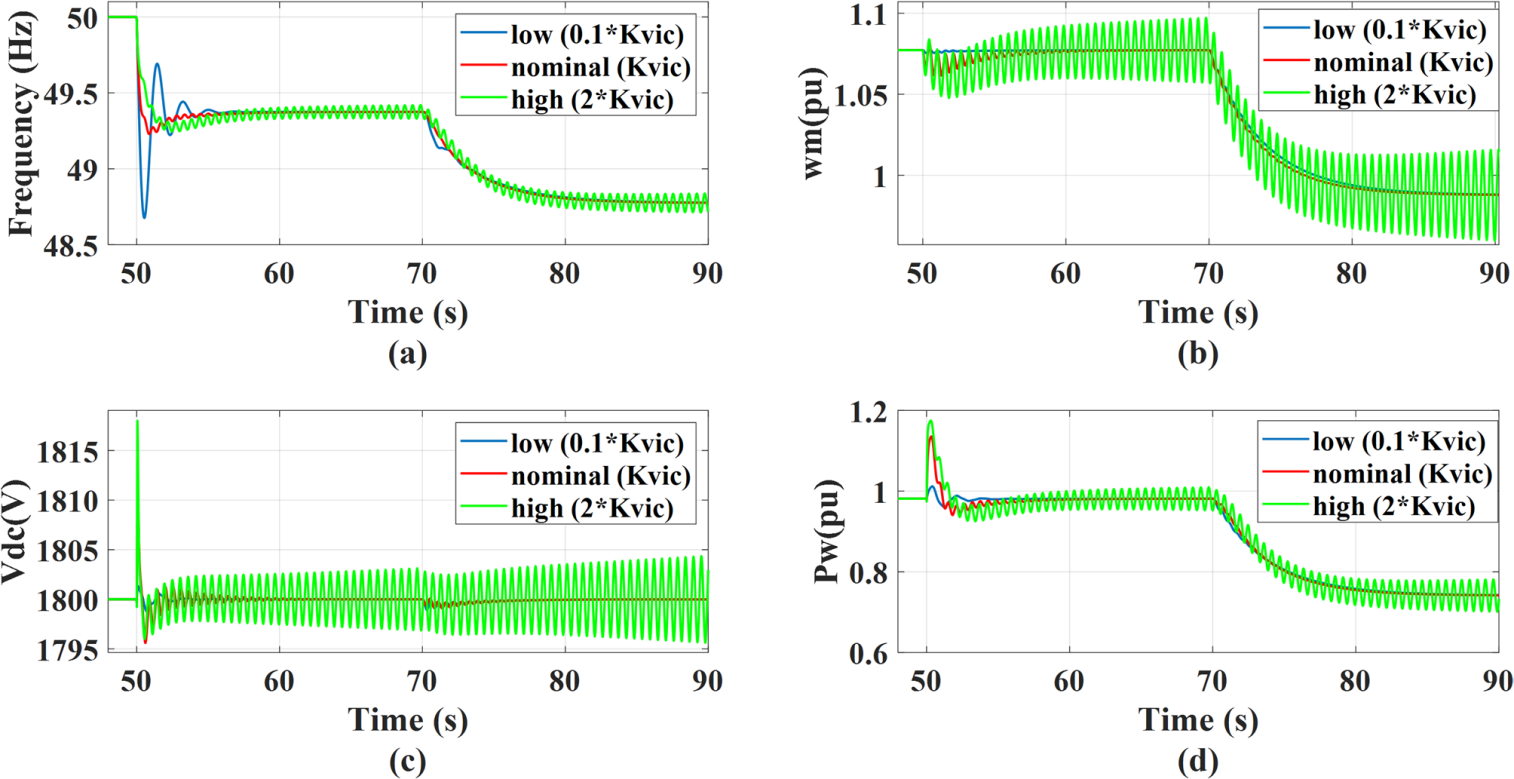
The system behavior response during load disturbance and wind speed change at different virtual inertia gains: (a) Grid frequency regulation, (b) Wind rotational speed response, (c) Response of the dc link voltage, and (d) Wind power response.

**Fig. 19:**
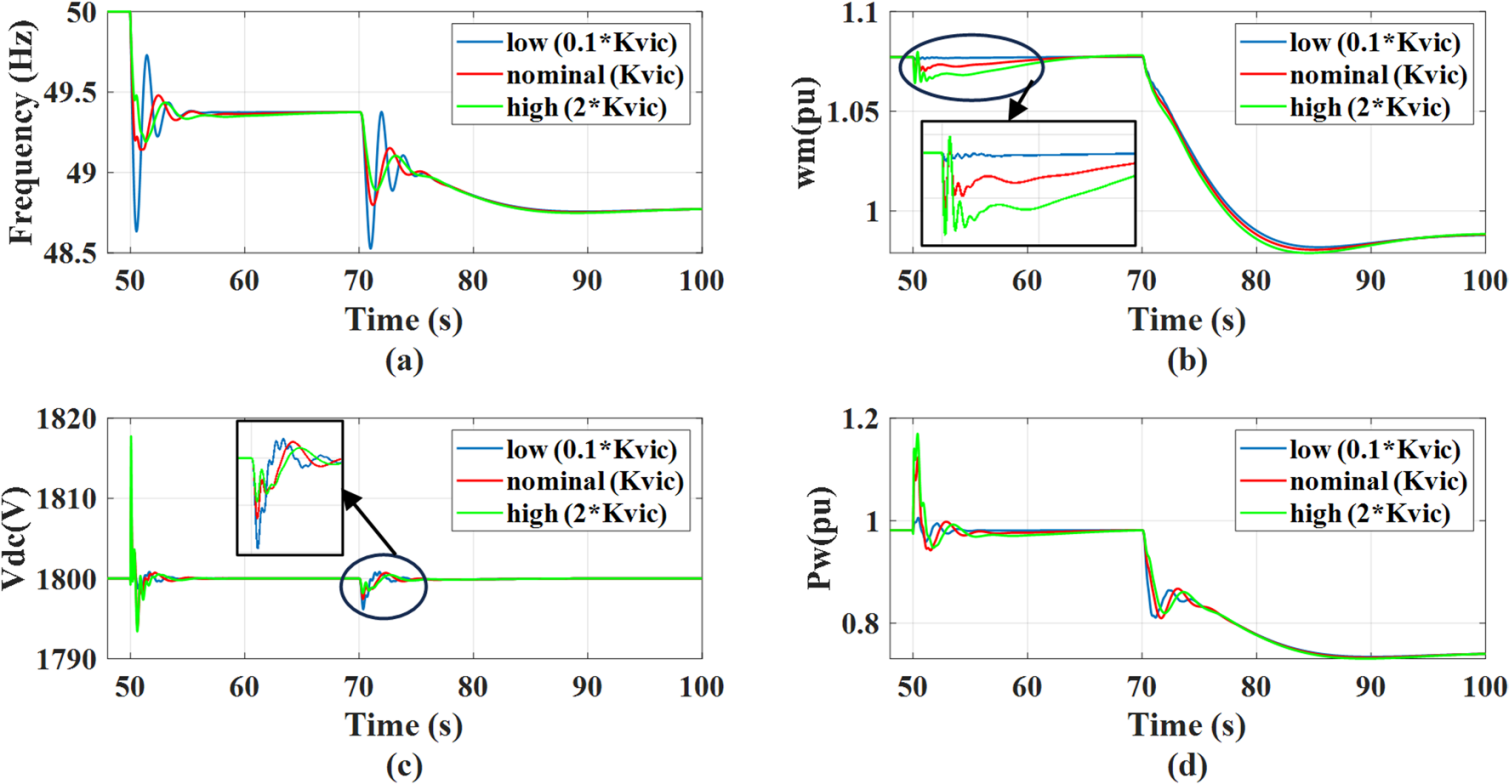
The system behavior response during load disturbance and wind speed change at different virtual inertia gains with adding outer damping loop: (a) Grid frequency response, (b) Wind rotational speed response, (c) DC link voltage response, and (d) Wind power response.

The system’s response is evaluated for virtual inertia gain values set to 0.1, 1, and 2 times the nominal value, under a 0.5 MW step change in AC static load occurring at *t* = 50s, followed by a wind speed drop from 12 m/s to 11 m/s at *t* = 70 s. A drop in wind speed fundamentally reduces the mechanical power captured by the PMSG, the MPPT control forces a reduction in power output, causing the rotor speed to decelerate until it aligns with the new lower optimal speed. The decrease in active power supplied to the grid creates a power deficit at PCC resulting in a reduction in grid frequency. Also, there will be a minor transient dip in dc link voltage before the GSC controller reacts to restore the reference value. Increasing the inertia gain improves the system frequency nadir during disturbance. However, Increasing the virtual inertia gain to high values leads to reduced damping and decreased system stability as the gain is two times the nominal value, the system will be unstable. This behavior is reflected in the responses of grid frequency, dc voltage, wind power, and rotational speed as shown in the Fig. 18. With adding the outer damping loop, the system will be more stable and greater damping, as shown in Fig. 19. Therefore, the system transitions from an unstable to a stable region when the virtual inertia gain is doubled relative to its nominal value.

### Shaft stiffness

**Fig. 20:**
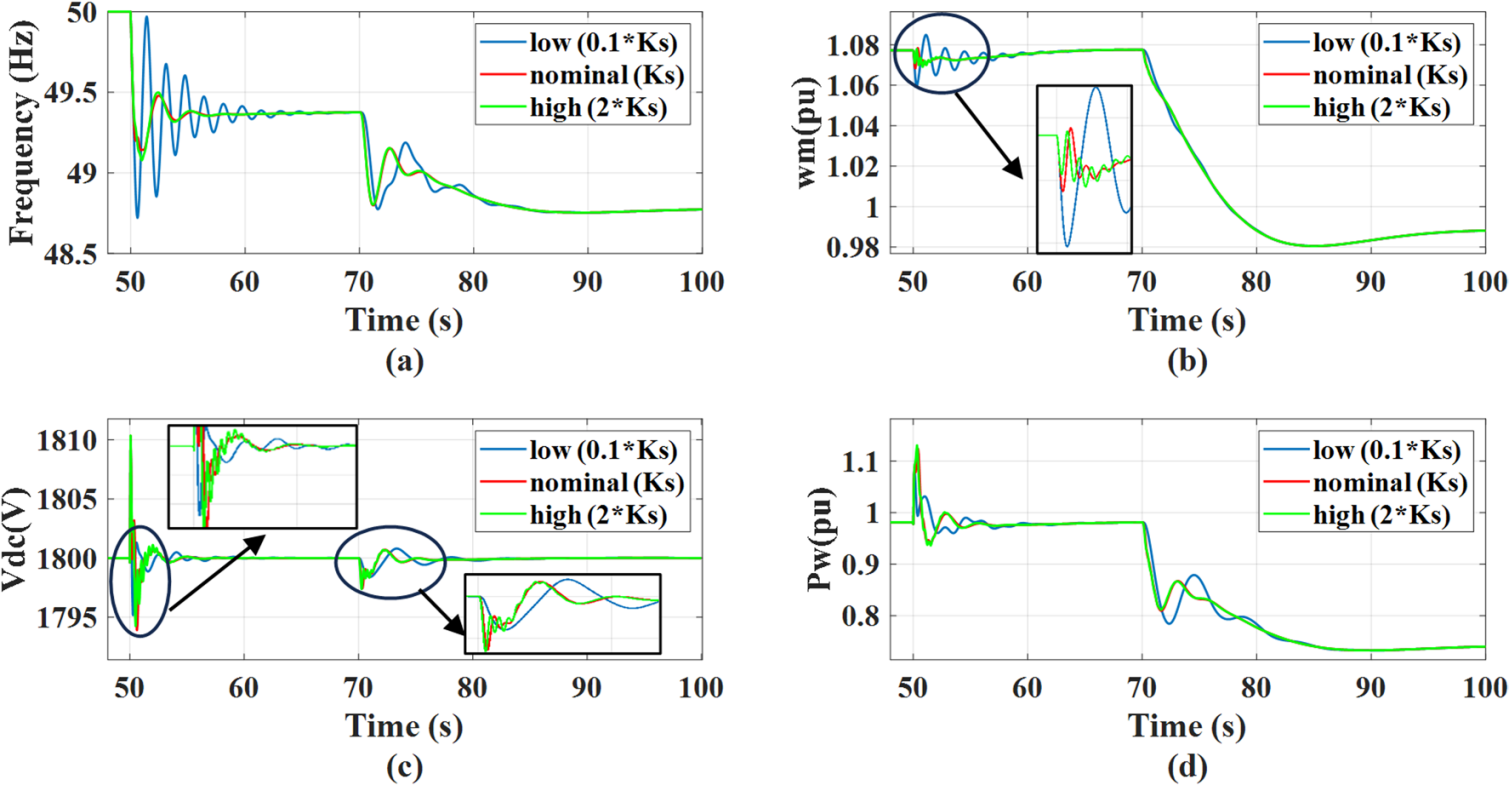
The system behavior response during load disturbance and wind speed change at different shaft stiffness gains with activating outer damping power: (a) Grid frequency response, (b) Wind rotational speed response, (c) DC link voltage response, and (d) Wind power response.

The system’s response is evaluated for shaft stiffness gain values set to 0.1, 1, and 2 times the nominal value, under a 0.5 MW step change in AC static load occurring at *t* = 50s, followed by a wind speed drop from 12 m/s to 11 m/s at *t* = 70 s. The shaft stiffness is a critical parameter that dictates the trade-off between mechanical stress limitations and electrical system stability during transient events. As concluded by prior eigenvalue analysis, the system may operate in an unstable region when characterized by low shaft stiffness and lacks supplementary damping. The introduction of outer damping power successfully shifts the system into a stable region; however, the resulting transient behavior and overall dynamics remain dependent on the specific shaft stiffness value. As shown in Fig. 20, during the load disturbance, lower shaft stiffness causes a significant mechanical decoupling between the PMSG rotor and the turbine shaft, resulting in large rotating speed swings, oscillatory in output power, and dc link voltage. These oscillations, particularly when interacting with virtual inertia control, propagate to the grid resulting in poor frequency damping. However, increasing shaft stiffness shifts the system’s mechanical natural frequency higher, making the system more rigid. This rigidity causes the mechanical modes to interact with the higher-frequency dynamics of the power converter control loops, resulting in high-frequency ripple in both the rotating speed and the DC link voltage. Although during the wind speed change, a decrease in wind speed is a relatively slow mechanical disturbance that causes a gradual reduction in mechanical torque, a stiffer shaft allows a smaller torsional angle between the turbine and the generator mass which reduces the amplitude of torsional oscillation that may be exciting during the deceleration process.

### Outer loop bandwidth

**Fig. 21:**
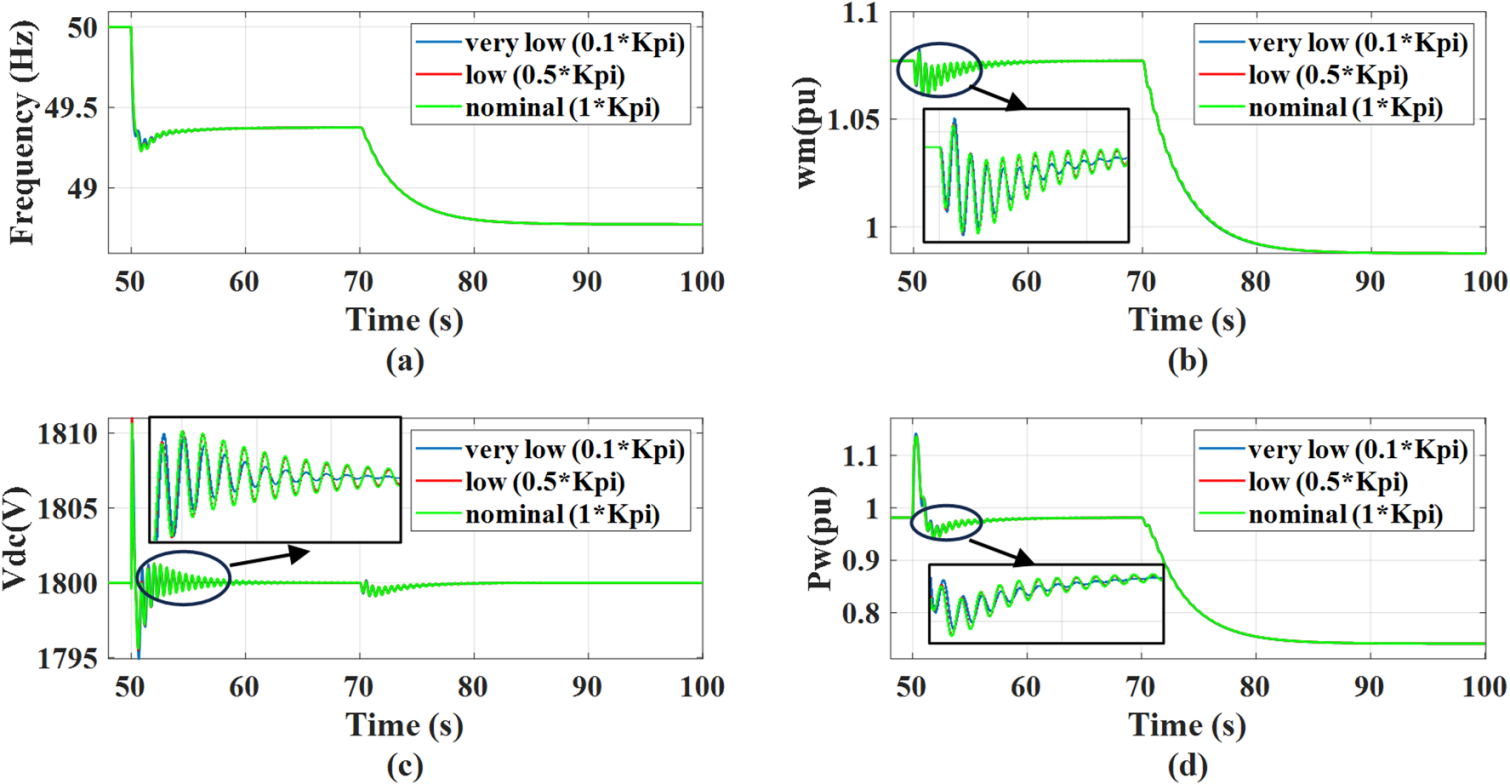
The system behavior response during load disturbance and wind speed change at different MSC integral gains with deactivating outer damping power: (a) Grid frequency response, (b) Wind rotational speed response, (c) DC link voltage response, and (d) Wind power response.

**Fig. 22:**
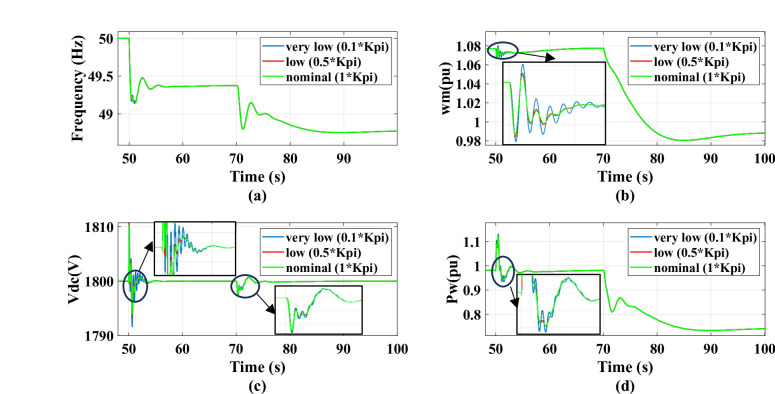
The system behavior response during load disturbance and wind speed change at different MSC integral gains with activating outer damping power: (a) Grid frequency response, (b) Wind rotational speed response, (c) DC link voltage response, and (d) Wind power response.

**Fig. 23:**
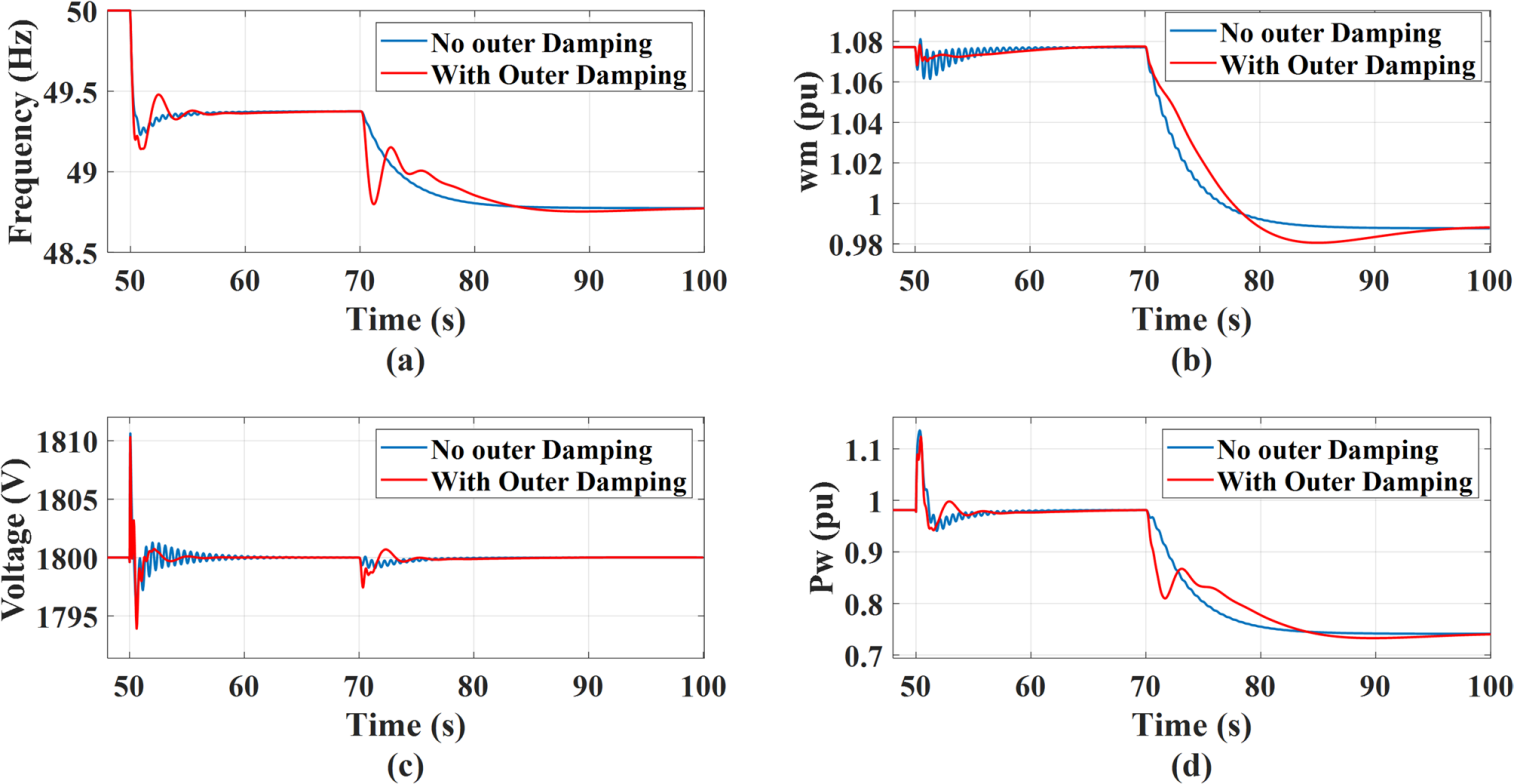
Comparison between system response during load disturbance and wind speed change with and with no outer damping loop: (a) Grid frequency, (b) Wind rotational speed, (c) DC link voltage response, and (d) Wind power response.

**Fig. 24:**
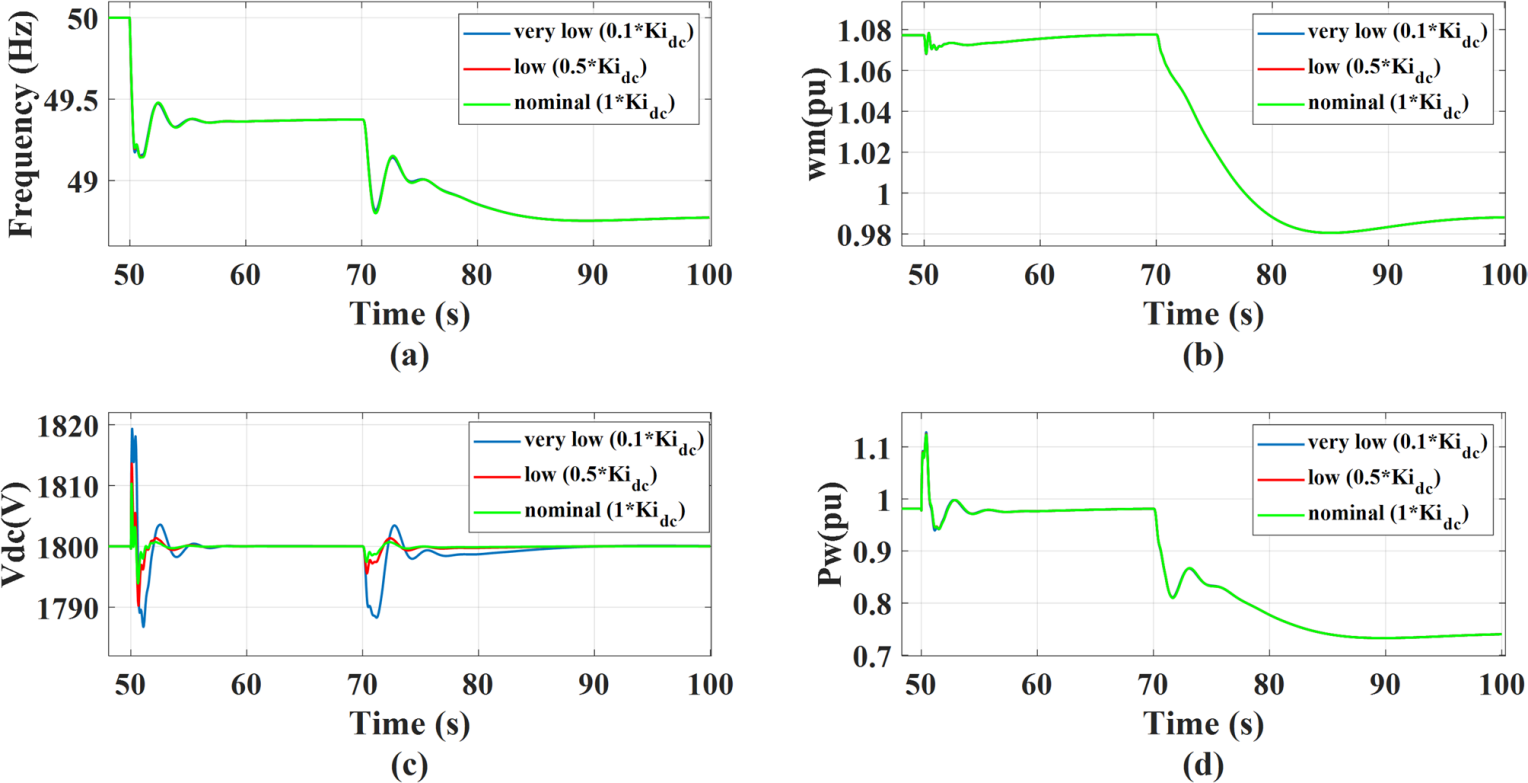
The system behavior response during load disturbance and wind speed change at different GSC integral gains with activating outer damping power: (a) Grid frequency response, (b) Wind rotational speed response, (c) DC link voltage response, and (d) Wind power response.

The system’s response is evaluated for integral gains of MSC and GSC values set to 0.1, 0.3, and 1 of the design values, under a 0.5 MW step change in AC static load occurring at *t* = 50s, followed by a wind speed drop from 12 m/s to 11 m/s at *t* = 70 s. The integral gain of the MSC is critical when the MSC is the primary controller compensating for electrical disturbances, such as load changes that require the MSC to aid in grid frequency regulation. However, its influence is minimal during mechanical disturbances, like a wind speed decrease, where the system response is dominated by mechanical inertia and the faster action of the GSC control. Thus, during a load disturbance, increasing the integral gain of the MSC active power controller effectively eliminates steady-state error of output power and rotational speed. It also risks increasing the control bandwidth, causing the aggressive power reference tracking to interact directly with the mechanical natural frequency, decreasing stability. This effect is not pronounced during a wind speed change, where the system dynamics are less sensitive to this parameter, as illustrated in Fig. 21. To overcome this negative impact on system stability, the inclusion of an outer damping loop significantly improves the system’s transient performance where it moves the system from an unstable region to a fully controllable one as shown in Fig. 23. Existing of damping makes the system achieve faster recovery and exhibits minimal oscillations in DC link voltage and rotor speed following a disturbance. This allows the use of a higher integral gain, which in turn give a better system performance and Faster Recovery with a minimal dip in the dc link voltage during disturbances in addition better grid support as demonstrated in Fig. 22. Although, increasing the integral gain of the GSC outer loop dc-link voltage controller has a direct impact on the dc link dynamic response as shown in Fig. 24, where the dc link voltage returns to its reference faster after disturbances. So, a higher integral gain improves the accuracy of dc-link voltage regulation by reducing steady state error and accelerating voltage recovery following disturbances. However, the GSC does not regulate grid frequency, rotor rotational speed, or the wind turbine’s active power output. Consequently, variations in the GSC integral gain do not have a direct impact on these quantities.

## Conclusion

This work employs a comprehensive linearized state-space model for a PMSG-based system integrated into a low-inertia AC/DC grid, yielding crucial stability insights. The eigenvalue-based stability analysis revealed the critical influence of different system parameters: Virtual Inertia Gain was shown to be effective in enhancing overall system damping, whereas low Shaft Stiffness and insufficient Grid Inertia significantly compromise stability margins and damping. Furthermore, the outer loop power control bandwidth was identified as a critical factor, demonstrating a necessary trade-off: faster bandwidths (higher gain) improve active power tracking but significantly reduce the system damping. The study confirms that adding outer loop damping substantially solves the majority of these damping problems, allowing the system to achieve both high-performance tracking and robust stability margins. These findings precisely quantify the stability margins and damping characteristics, providing essential guidance for controller tuning in low-inertia hybrid grid applications. Finally, with the help of MATLAB/SIMULINK, time domain non-linear simulations, justified the stability analysis carried out, confirming the correctness and the accuracy of the study.

## Data Availability

The datasets used and/or analyzed during the current study available from the corresponding author on reasonable request.
